# DHODH inhibition suppresses cutaneous squamous cell carcinoma growth by the induction of differentiation through perturbation of the cellular redox balance

**DOI:** 10.1038/s41419-026-08815-w

**Published:** 2026-04-28

**Authors:** Ferial Khalife, Elodie Muzotte, Fatima Naji, Walid Mahfouf, Julien Izotte, Benoît Pinson, Stéphane Claverol, Nivea Amoedo, Hussein Fayyad-Kazan, Lea Dousset, Rodrigue Rossignol, Hamid-Reza Rezvani

**Affiliations:** 1 Univ. Bordeaux, Inserm, BRIC, UMR 1312, Bordeaux, France; 2 Animalerie A2, University of Bordeaux, Bordeaux, France; 3 Metabolic Analyses Service, TBMCore-Université de Bordeaux-CNRS UAR 3427-INSERM, US005 Bordeaux, France; 4 Univ. Bordeaux, Bordeaux Proteome, Bordeaux, France; 5 Inserm U1211 Maladies Rares: Génétique et Métabolisme (MRGM), Université de Bordeaux, Bordeaux, France; 6 CELLOMET, Centre de Génomique Fonctionnelle de Bordeaux, Université de Bordeaux, Bordeaux, France; 7 Laboratory of Cancer Biology and Molecular Immunology, Faculty of Sciences I, Lebanese University, Hadath, Lebanon

**Keywords:** Cancer metabolism, Proteomics, Stress signalling

## Abstract

Dihydroorotate dehydrogenase (DHODH), a key enzyme in de novo pyrimidine biosynthesis, has recently emerged as a therapeutic target in various cancers. We have previously identified a pivotal role of DHODH in the initiation of cutaneous squamous cell carcinoma (cSCC), the second most common type of non-melanoma skin cancer. We also showed that pharmacological inhibition of this enzyme suppresses ultraviolet (UV)-induced tumor formation. However, the key mechanisms driving the anticancer activity of DHODH inhibition remain unexplored in cSCC. We investigated the biological consequences of pharmacological and genetic DHODH inhibition in cSCC using xenograft models derived from two human cell lines, A431 and SCC13, implanted in immunodeficient NSG mice. DHODH activity was suppressed pharmacologically with leflunomide (LFN) and the potent DHODH inhibitor PTC299, or genetically via lentiviral shRNA-mediated DHODH silencing (shDHODH). Proteomic and metabolomic analyses were integrated with histopathological, immunohistochemical, and immunoblotting evaluations to delineate the downstream effects of DHODH blockade. Comprehensive proteomic and metabolomic profiling revealed that DHODH inhibition induces a coordinated adaptive program involving keratinization, differentiation, redox homeostasis, and metabolic stress responses. Histological and immunostaining analyses demonstrated marked reductions in Ki67-positive proliferating cells and a corresponding increase in pan-cytokeratin (PanCK) and keratin 10 (Krt10) expression, indicative of enhanced epithelial differentiation. These changes were most pronounced in PTC299-treated and shDHODH xenografts, whereas LFN displayed minimal or no efficacy in SCC13 tumors. DHODH inhibition drives tumor differentiation and suppresses proliferation in cSCC, highlighting metabolic dependency as a potential therapeutic vulnerability. PTC299 exhibited superior antitumor activity and differentiation-inducing capacity compared with LFN. These findings position DHODH as a promising target for bioenergetic vulnerability-based cancer therapy in advanced or treatment-resistant cSCC.

## Introduction

Cutaneous squamous cell carcinoma (cSCC) is the second most common form of non-melanoma skin cancer, and its global incidence continues to rise [1]. While the majority of cSCCs can be managed effectively by surgical excision, a subset of tumors display aggressive biological behavior, with increased risks of local invasion, recurrence, and metastasis [2]. These high-risk lesions are particularly prevalent in immunosuppressed individuals, such as organ-transplant recipients, who exhibit up to a 65- to 250-fold higher incidence of developing skin cancer compared with the general population [3]. In such patients, conventional treatments, including surgery, radiotherapy, and systemic chemotherapy, are often less effective, and even novel immune-checkpoint inhibitors can show limited or transient benefit [4]. Despite advances in prevention and treatment, aggressive or metastatic cSCC remains a clinical challenge, underscoring the need to identify new therapeutic vulnerabilities.

Emerging evidence suggests that metabolic reprogramming is a critical feature of cSCC progression [5–9]. Tumor cells actively rewire metabolic networks to sustain rapid proliferation, favoring glycolysis and de novo nucleotide synthesis over oxidative metabolism [10]. Within this metabolic landscape, dihydroorotate dehydrogenase (DHODH) has emerged as a key regulatory enzyme at the crossroad between pyrimidine biosynthesis and mitochondrial energy transduction processes. DHODH is the mitochondrial flavoprotein that catalyzes the oxidation of dihydroorotate to orotate, the fourth and rate-limiting step in the de novo pyrimidine biosynthesis pathway [11]. This reaction is tightly coupled to mitochondrial respiration through the ubiquinone pool, thereby linking nucleotide synthesis with the electron transport chain and overall cellular bioenergetics [12]. Inhibition of DHODH has emerged as a promising therapeutic strategy to suppress tumor growth across multiple cancer types, such as triple-negative breast cancer [13], KRAS-mutant tumors [14], melanoma [15], glioma [16–18], and acute myeloid leukemia [19]. Several small-molecule DHODH inhibitors, such as leflunomide (LFN), brequinar, PTC299, and BAY2402234, have been investigated for their anticancer effects in preclinical tumor models and in selected clinical trials [20, 21]. Our group previously identified a pivotal role of DHODH in skin carcinogenesis. We demonstrated that ultraviolet-B (UVB) exposure upregulates DHODH expression in epidermal keratinocytes, and that pharmacological inhibition of this enzyme using the FDA-approved drug LFN suppresses UV-induced tumor formation in mouse models [7, 8]. Building on these findings, we recently evaluated the efficacy of LFN in patient-derived xenograft (PDX) models. Our results revealed a heterogeneous response among the PDXs, indicating that tumor sensitivity to LFN depends on the metabolic score of each cSCC tumor (Metscore_tumor_). Using metabolic profiling, we further identified three subgroups with low, medium, and high metabolic scores across all stages of carcinogenesis [5]. Notably, tumors with low metabolic scores responded more favorably to LFN, suggesting that the metabolic profile plays a key role in determining therapeutic response.

Although previous studies have shown that DHODH inhibition can exert antitumor effects through multiple mechanisms, including metabolic intervention, immune modulation, induction of ferroptosis or necroptosis, and regulation of cellular differentiation [20, 21], the principal mechanisms underlying the anticancer effects of DHODH inhibition have not yet been explored in cSCC. In the present study, we sought to systematically investigate the functional consequences of DHODH inhibition in vivo using xenograft models derived from two distinct human cSCC cell lines, A431 and SCC13, implanted in immunodeficient NSG mice. DHODH activity was targeted through both pharmacological agents (LFN and PTC299, a potent and selective DHODH inhibitor) and a genetic approach involving a lentiviral vector expressing shRNA-mediated silencing of DHODH. Comprehensive proteomics and metabolomics were then performed to elucidate the molecular pathways altered upon DHODH inhibition and to identify mechanisms underlying differential treatment responses. Together, our findings reveal that DHODH inhibition suppresses tumor growth, promotes keratinocyte differentiation, and disrupts pyrimidine biosynthesis and mitochondrial metabolism. These results position DHODH as a critical metabolic node in cSCC and support its potential as both a therapeutic target and a predictive biomarker for metabolic therapy in skin cancer.

## Materials and methods

### Cell culture

A431, a cell line isolated from an 85-year-old female patient with epidermoid carcinoma, was obtained from ATCC. SCC13, a cell line isolated from a 56-year-old female patient with epidermoid carcinoma, was kindly provided by Dr. James Rheinwald [22]. These cells were cultured in DMEM media (4.5 g/L D-Glucose, GlutaMAXTM, pyruvate; Gibco) supplemented with 10% fetal bovine serum (FBS) and 1% penicillin-streptomycin. Cells were maintained at 37 °C in a humidified atmosphere containing 5% CO₂. Both cell strains were cryopreserved within three passages, and no cell aliquot was cultured continuously for more than 6 months. A431 and SCC13 cell cultures were tested every 2 weeks for mycoplasma contamination by PCR and always came back negative.

### In vivo xenograft

NOD/Shi-SCID IL2Rγnull mice (NSG) were bred in standard conditions compliant with regulations and housed in a pathogen-free animal facility. Female mice were used in all experiments. Experimental subgroups consisted of 10 mice per group, with each group of 5 mice caged separately. Cells were combined with Matrigel® Matrix High Concentration (Corning, USA) and subcutaneously injected into the right flank of mice. For A431 cells, we used 20,000 cells per injected site, and for SCC13, we used 50,000 cells per injected site (one injection per mouse). When the tumors reached approximately 200 mm^3^ in volume, mice were randomly assigned to different groups and experiments were performed blinded with respect to treatment. Treatments consisted of LFN (oral gavage of 35 mg/kg/day), PTC299 (oral gavage of 10 mg/kg/day), or Carboxymethyl cellulose (CMC, oral gavage as vehicle). To assess the tumor volumes and growth rate of tumors, caliper measurements of the tumors were performed twice a week. Tumor volumes were calculated by the following formula: volume = (width)^2^ × length/2. Mice injected with A431 or SCC13 cells were sacrificed after 27–35 days or 65–78 days of treatment, respectively, and their organs were analyzed macroscopically. The mice were euthanized by cervical dislocation to prevent suffering. Photos of the tumors were taken, and the tumors were then sectioned into three parts: one part was embedded in paraffin for histological analyses, and the remaining two were directly frozen in liquid nitrogen and stored at –80 °C for Western blot, proteomic, and metabolomic analyses. All mouse experiments were conducted in accordance with institutional guidelines and were approved by the Bordeaux University Animal Care and Use Committee, in compliance with applicable national and international regulations.

Additional materials and methods are provided in additional Supplementary Material file (Appendix S1).

## Results

### PTC299 more effectively targets DHODH and suppresses tumor growth than leflunomide

To evaluate the impact of DHODH inhibition on cSCC growth, we employed three complementary approaches: (1) pharmacological inhibition of DHODH in mice bearing xenografts of A431 and SCC13 human cells through oral administration of LFN or PTC299 (PTC), and (2) genetic silencing of DHODH via transduction of cells with a lentiviral vector expressing shRNA targeting DHODH (shDHODH) prior to transplantation into NSG mice.

**Fig. 1:**
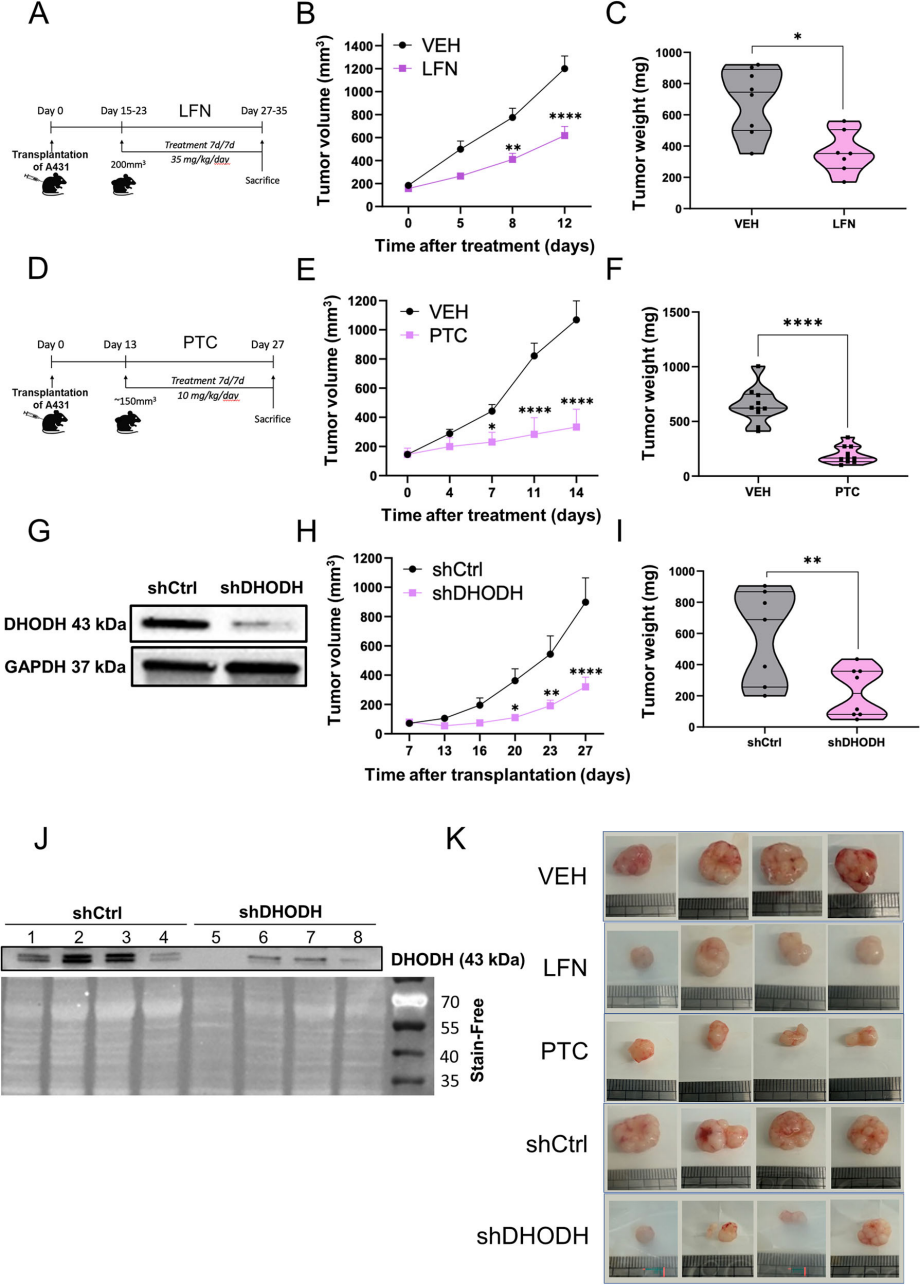
Pharmacological (LFN, PTC299) and genetic (shDHODH) inhibition of DHODH suppresses cSCC tumor growth in A431 xenograft models. **A**–**C** Schematic overview of the experimental design. A431 cells were transplanted into NSG mice, and treatment was initiated once tumors reached a volume of approximately 200 mm³. Mice received oral administration of either leflunomide (LFN) or vehicle (carboxymethylcellulose, VEH) for 12 days (**A**). Tumor growth was monitored throughout the treatment period. Growth curves show a significant reduction in tumor volume in the LFN-treated group compared with VEH controls (**B**). Final tumor weights at the experimental endpoint confirm reduced tumor burden following LFN treatment (**C**). *n* = 8 mice for VEH and *n* = 7 mice for LFN. **D**–**F** Experimental design for PTC299 treatment (10 mg/kg/day). After A431 cell transplantation, treatment commenced when tumors reached approximately 150 mm³. Mice were orally administered PTC299 or carboxymethylcellulose (vehicle, VEH) (**D**). Tumor growth curves demonstrate a significant reduction in tumor volume in the PTC299-treated group compared with VEH controls (**E**). Final tumor weights confirm strong inhibition of tumor growth by PTC299 (**F**). *n* = 10 mice per group. **G**–**J** A431 cells were transduced with lentiviral vectors expressing either shControl (shCtrl) or shDHODH. Western blot analysis confirmed efficient DHODH knockdown in A431 cells transduced with shDHODH prior to xenograft implantation (**G**). Following transplantation, tumor growth was measured and monitored over time (**H**). Tumor weights at sacrifice demonstrate a significant reduction in tumor burden in the shDHODH group compared with shCtrl (**I**). *n* = 7 mice for shCtrl and *n* = 8 mice for shDHODH. Western blot analysis of four shDHODH and four shCtrl xenografts confirmed sustained DHODH suppression in shDHODH tumors at the experimental endpoint, with a stain-free gel used for normalization (**J**). **K** Representative macroscopic images of excised tumors from each treatment group (VEH, LFN, PTC299, shCtrl, shDHODH). Data are presented as mean ± SEM. Statistical significance for tumor growth curves was determined using two-way ANOVA with Sidak’s post hoc test. Tumor weight comparisons at endpoint were analyzed using an unpaired two-tailed Student’s *t* test (*p* < 0.05, *p < 0.01, ****p < 0.0001).

After transplantation of A431 cells and once tumors reached an average volume of 200 mm^3^, mice were divided in two groups receiving either the vehicle alone or LFN administrated orally on a daily basis (Fig. 1A). Tumor growth was monitored periodically (Fig. 1B), and the tumors were weighted at the time of resection (Fig. 1C). The same procedure was applied for treatment with PTC (Fig. 1D–F). Both LFN and PTC treatments were well-tolerated, as indicated by stable body weight (Fig. S1A) and the absence of behavioral differences among groups. Both drugs significantly reduced tumor growth (Fig. 1B, E) and tumor weight (Fig. 1C, F) compared with their corresponding vehicle-treated controls. To further investigate DHODH targeting approaches, DHODH expression was downregulated in A431 cells using shDHODH. Western blot analysis confirmed robust DHODH downregulation in shDHODH-transduced A431 cells (Fig. 1G). Six days after transduction, the cancer cells were subcutaneously engrafted into NSG mice. Tumor growth kinetics (Fig. 1H) and final tumor weight (Fig. 1I) were significantly reduced in the shDHODH group compared with the control group transduced with non-targeting shRNA (shCtrl). Western blot analysis at the endpoint confirmed persistent DHODH downregulation in shDHODH-transduced tumors (Fig. 1J). Among the three tested treatment strategies, PTC299 produced the most pronounced effect, leading to a marked suppression of tumor growth and a significant reduction in final tumor weight (Fig. 1K). Together, these results demonstrate that both pharmacological and genetic DHODH inhibition effectively impair A431 tumor growth in vivo, with PTC299 exhibiting the strongest antitumor activity.

**Fig. 2:**
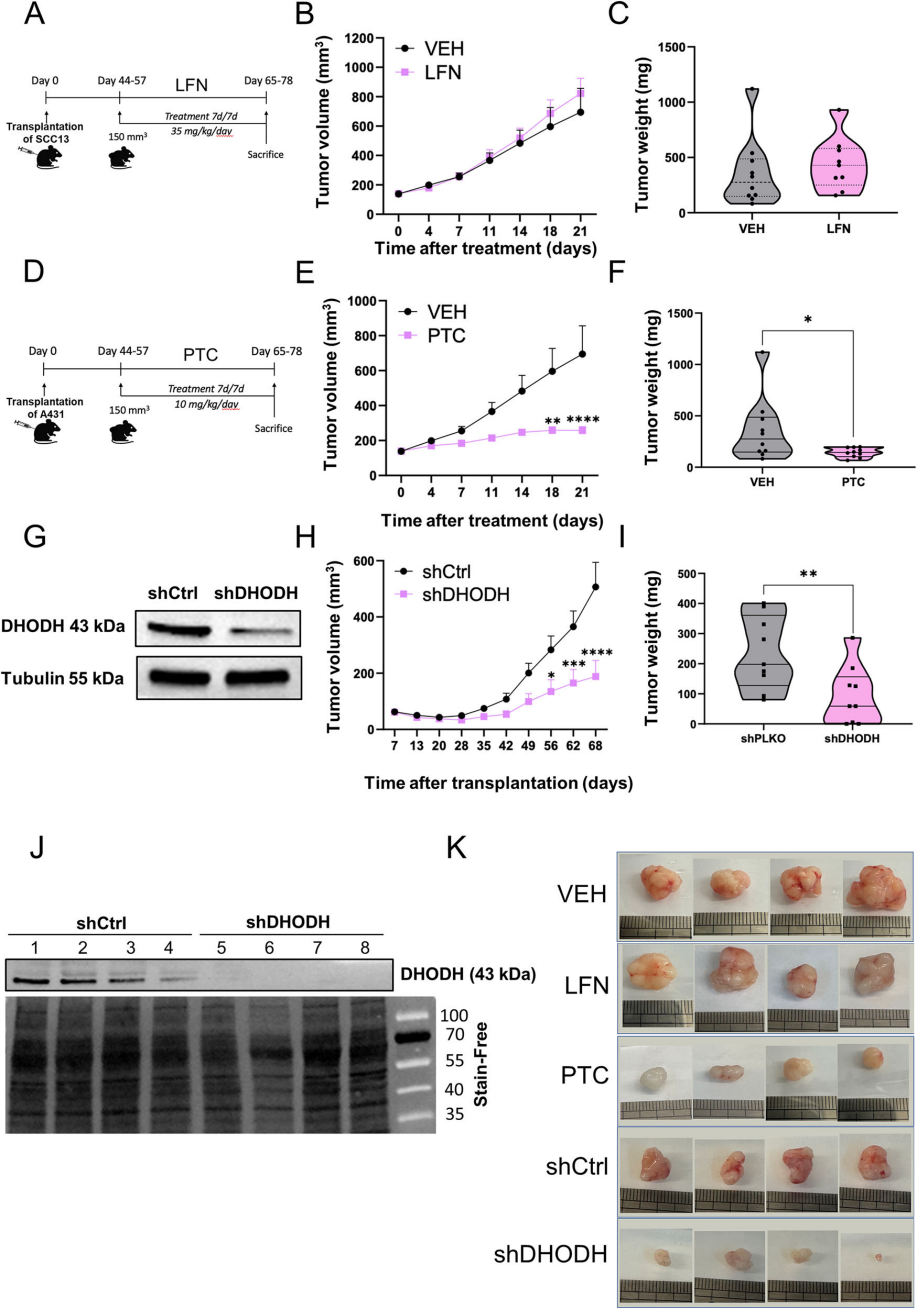
PTC299 and genetic DHODH silencing inhibit SCC13 xenograft growth, whereas LFN shows no efficacy. **A**–**C** Experimental timeline for SCC13 xenografts treated with LFN (35 mg/kg/day for 21 days). SCC13 cells were transplanted into NSG mice, and treatment was initiated once tumors reached approximately 150 mm³ (**A**). Tumor growth was monitored throughout the treatment period. Growth curves show no significant reduction in tumor volume in the LFN-treated group compared with VEH controls (**B**). Final tumor weights at the experimental endpoint confirm the absence of response to LFN in SCC13 tumors (**C**). *n* = 10 mice per group. **D**–**F** Experimental design for PTC299 treatment (10 mg/kg/day for 21 days). Following tumor establishment (~150 mm³), mice received oral administration of either PTC299 or VEH (**D**). Tumor growth curves demonstrate a significant reduction in tumor volume in the PTC-treated group compared with VEH controls (**E**). Final tumor weights at sacrifice confirmed marked inhibition of tumor growth by PTC299 (**F**). *n* = 10 mice per group. **G**–**J** SCC13 cells were transduced with lentiviral vectors expressing either shCtrl or shDHODH. Western blot confirmed efficient DHODH downregulation in shDHODH-transduced SCC13 cells prior to transplantation into NSG mice (**G**). Tumor growth monitoring revealed a significant reduction in tumor volume in the shDHODH group compared with shCtrl (**H**). Final tumor weights corroborate a significant decrease in tumor burden in the shDHODH group (**I**). Western blot analysis of xenograft samples at the experimental endpoint confirmed sustained DHODH downregulation in shDHODH tumors (**J**). **K** Representative macroscopic images of excised tumors from each treatment group (VEH, LFN, PTC, shCtrl, shDHODH). Data are presented as mean ± SEM. Statistical significance of tumor growth curves was determined by two-way ANOVA with Sidak’s post hoc test. Tumor weight comparisons at endpoint were analyzed using an unpaired two-tailed Student’s *t* test (*p < 0.05, **p < 0.01, ***p < 0.001, ****p < 0.0001).

To determine whether DHODH inhibition exerts similar effects in a second human cSCC model, SCC13 xenografts were subjected to the same treatment protocols (Figs. 2A–K and S1B). In contrast to A431 tumors, LFN treatment did not significantly affect SCC13 tumor growth (Fig. 2B) or final tumor weight (Fig. 2C), indicating that this cell line was relatively resistant to LFN. Conversely, both PTC299 administration and shDHODH expression markedly reduced tumor growth and final tumor mass (Fig. 2D–I, K), confirming potent inhibition of tumor proliferation.

Collectively, these findings indicate that DHODH inhibition by PTC or shDHODH technology suppresses tumor growth in both cSCC models, whereas LFN is ineffective in SCC13 tumors. PTC299 thus emerges as a more potent DHODH-targeting compound with stronger antitumor activity.

### Differential sensitivity of cSCC cell lines to DHODH inhibition in vitro

**Fig. 3:**
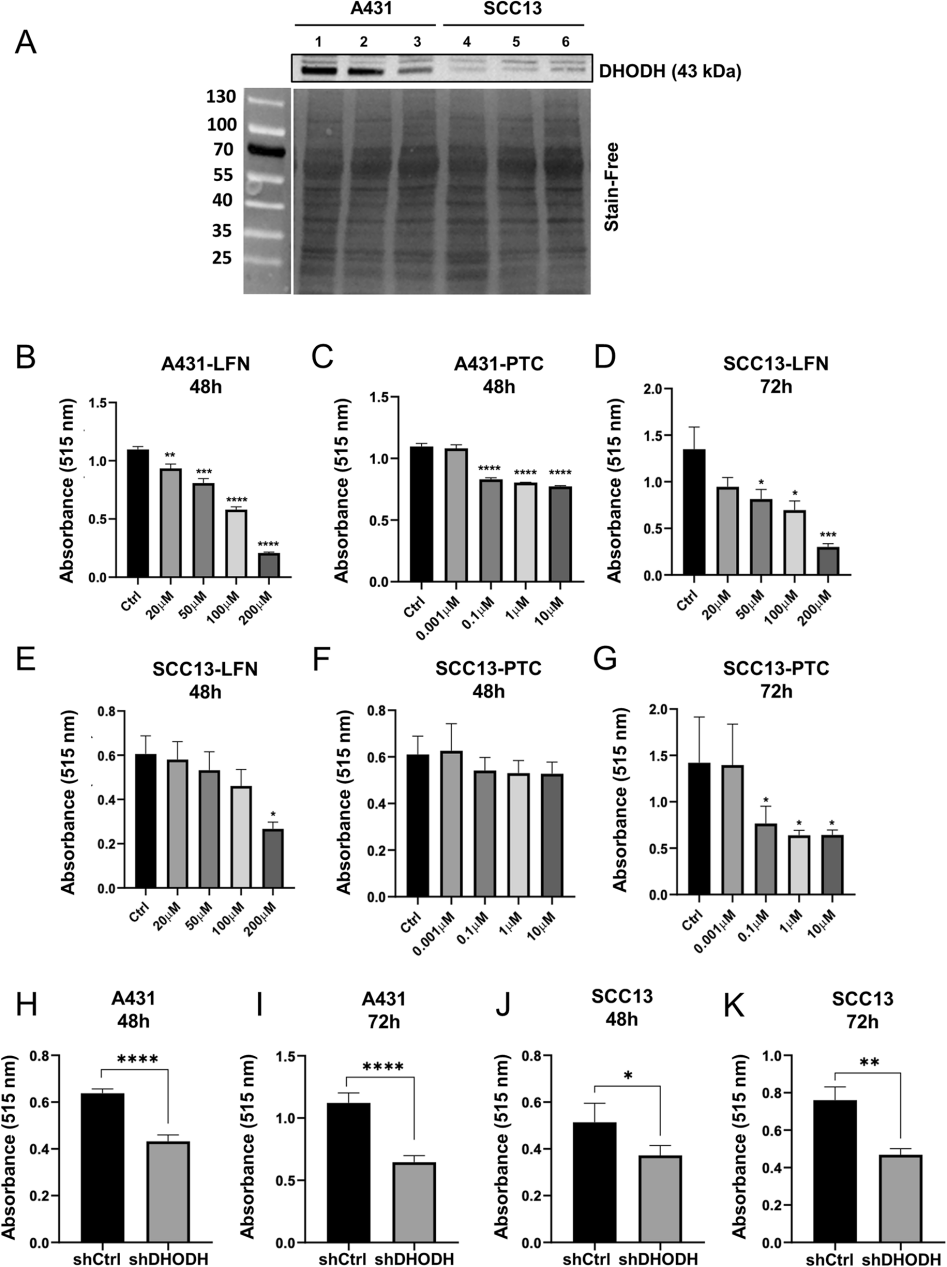
A431 cells exhibit higher DHODH expression and greater susceptibility (sensitivity) to DHODH inhibition than SCC13 cells. **A** Western blot analysis of DHODH expression in vehicle-treated A431 and SCC13 xenograft samples. A431 tumors exhibited higher DHODH protein levels than SCC13. A stain-free gel was used as a loading control. **B**−**G** Effects of leflunomide (LFN) and PTC299 on cell viability in A431 (**B**, **C**) and SCC13 (**D**–**G**) cells assessed by SRB assays. Cells were seeded in 96-well plates and treated with the indicated concentrations of LFN or PTC299 for 48 or 72 h. Both inhibitors reduced cell viability in a dose-dependent manner, with A431 cells showing a more pronounced response at lower concentrations, whereas SCC13 cells were comparatively less sensitive. **H**–**K** Lentiviral shRNA-mediated DHODH downregulation (shDHODH) significantly reduced cell viability in both A431 and SCC13 cell lines compared with shCtrl at 48 and 72 h. Data represent mean ± SD of at least three independent experiments performed in triplicate. Statistical significance was determined by one-way ANOVA with Dunnett’s post hoc test versus control (*p < 0.05, **p < 0.01, ***p < 0.001, ****p < 0.0001).

Since we previously demonstrated that the sensitivity of PDXs to LFN treatment depends on DHODH expression levels [5], we hypothesized that the different responses to LFN observed between A431 and SCC13 xenografts might result from differences in their basal DHODH expression. To test this hypothesis, we assessed DHODH expression in vehicle-treated A431 and SCC13 xenografts. The results showed that A431 tumors exhibited higher DHODH expression compared to SCC13 (Fig. 3A), suggesting a greater reliance on de novo pyrimidine synthesis and an increased susceptibility to DHODH inhibition.

To further investigate the cellular effects of DHODH inhibition, A431 and SCC13 cells were exposed to increasing concentrations of LFN or PTC299. In proliferation assays, A431 cells displayed a dose-dependent reduction in viability following treatment with either compound (Fig. 3B, C), whereas SCC13 cells showed a delayed response, with significant effects observed only after 72 h of exposure (Fig. 3D–G). Moreover, A431 cells were sensitive to LFN at lower doses compared with SCC13, consistent with the higher DHODH expression (Fig. 3B, D, A). Genetic silencing of DHODH using shDHODH transduction significantly decreased cell viability in both lines at 48 and 72 h (Fig. 3H–K), confirming that DHODH loss impairs tumor cell proliferation.

To determine whether the reduction in cell viability induced by DHODH inhibition was associated with altered cell-cycle progression or apoptotic cell death, we performed cell-cycle analysis using propidium iodide (PI) staining in combination with EdU incorporation. Flow cytometry analysis revealed a pronounced accumulation of cells in the S phase following treatment with the DHODH inhibitor PTC299, accompanied by a significant reduction in the proportion of cells in the G0/G1 phase (Fig. S2). These results indicate that DHODH inhibition primarily induces S-phase arrest, suggesting impaired DNA replication rather than apoptosis as the major contributor to reduced cell proliferation.

Together, these results indicate that DHODH inhibition reduces in vitro cSCC cell survival in a time- and cell line–dependent manner, with A431 cells exhibiting greater sensitivity due to higher DHODH expression.

### Metabolomic profiling reveals metabolism reprogramming and redox imbalance following DHODH inhibition

**Fig. 4:**
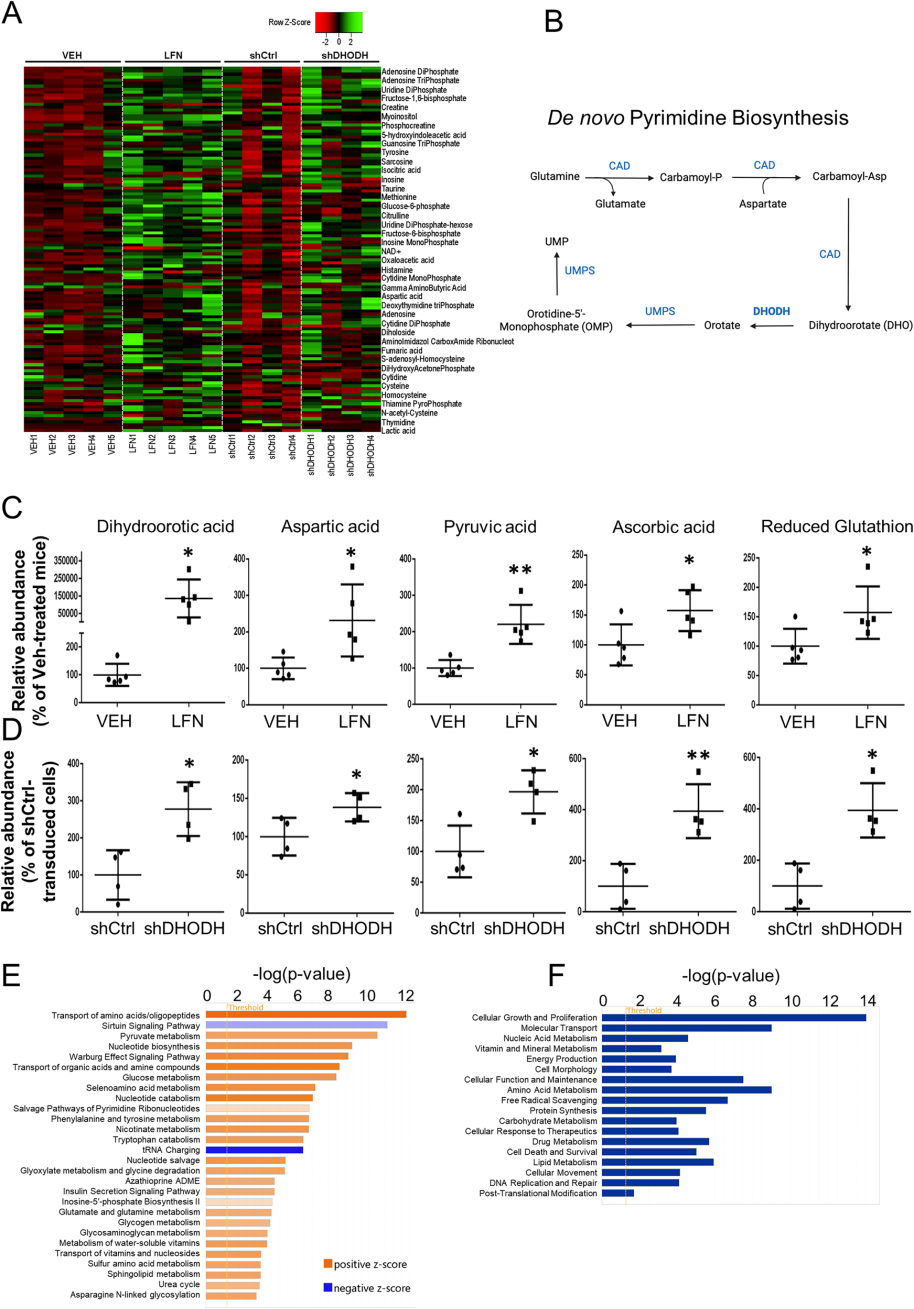
DHODH inhibition triggers pyrimidine precursor accumulation, metabolic rerouting, and oxidative stress responses. **A**–**D** Semi-targeted metabolomic profiling was performed on A431 xenografts treated with LFN or VEH (*n* = 5 per group), and on samples from shCtrl and shDHODH groups (*n* = 4 per group). Heatmap illustrating differential metabolite abundances across conditions. Colors represent low (red) and high (green) metabolite levels (**A**). Schematic of the de novo pyrimidine biosynthesis pathway. CAD carbamoyl-phosphate synthetase 2, aspartate transcarbamylase, and dihydroorotase, DHODH DHO dehydrogenase, UMPS uridine 5’-monophosphate synthase (**B**). Relative abundances of dihydroorotic acid, aspartic acid, pyruvic acid, ascorbic acid, and reduced glutathione in LFN-treated vs. vehicle xenografts (**C**) and in shDHODH vs. shCtrl samples (**D**). (Data represent mean ± SD for *n* = 4–5 biological replicates. Statistical significance was assessed by multiple *t*-tests with *p* < 0.05 and FDR 1% (*p < 0.05, **p < 0.01). **E** Ingenuity Pathway Analysis (IPA®) of differentially abundant metabolites, highlighting the top significantly affected canonical pathways (*z*-score >2). The dashed line denotes the significance threshold (−log(*p*) = 1.3). **F** Gene Ontology enrichment analysis of the differentially expressed metabolites shows significantly enriched molecular and cellular functions. Only GO terms that were significantly overrepresented (*p* < 0.05) are shown.

To explore the metabolic consequences of DHODH inhibition, semi-targeted metabolomic profiling was performed on A431 xenografts treated with either LFN or vehicle (*N* = 5), as well as on samples from shCtrl and shDHODH (*N* = 4) groups (Fig. 4). Quantitative comparison of the 122 detected metabolites revealed significant differences in metabolites *Z*-scores between treatment groups, indicating broad metabolic alterations upon DHODH inhibition (Fig. 4A). Of note, hierarchical clustering of these metabolites was performed using Heatmapper (Average linkage; Euclidean distance). The comparative analysis of individual metabolites showed significant alterations in key intermediates of the de novo pyrimidine biosynthetic pathway and redox metabolism (Fig. 4B, C). Notably, dihydroorotic acid, the direct substrate of DHODH (Fig. 4B), accumulated following both pharmacological and genetic inhibition, confirming effective blockade of DHODH activity (Fig. 4C, D). In parallel, aspartic acid and pyruvic acid levels were significantly increased, suggesting metabolic rerouting toward glycolysis and amino acid metabolism (Fig. 4C, D). In addition, elevated levels of ascorbic acid and reduced glutathione (GSH) were detected in DHODH inhibition condition (Fig. 4C, D), suggesting activation of antioxidant defense mechanism and heightened redox stress in response to DHODH suppression.

Differentially abundant metabolites were further analyzed using the Ingenuity Computational Pathway Analysis (IPA®) software (www.ingenuity.com) to identify the molecular pathways and cellular functions affected by DHODH inhibition. As shown in Fig. 4E, the most dysregulated canonical pathways (*z*-score >2) included pyruvate metabolism, nucleotide metabolism, glucose metabolism, amino acid metabolism, and nicotinate metabolism, highlighting substantial reprogramming of energy metabolism upon DHODH inhibition. Functional enrichment analysis revealed that cellular growth and proliferation, energy metabolism, and redox balance homeostasis were among the most affected processes (Fig. 4F).

Semi-targeted metabolomic profiling was also performed on A431 xenografts treated with PTC299 or vehicle (*n* = 5). Among 159 analyzed metabolites, 48 were significantly altered upon DHODH inhibition (Fig. S3A, B), confirming broad metabolic remodeling. Consistent with the results obtained with LFN, comparative analysis revealed marked alterations in intermediates of the de novo pyrimidine biosynthesis pathway, including dihydroorotate, UTP, CTP, dCTP, and dTTP (Fig. S3B, C). In particular, a profound accumulation of dihydroorotate ( > 330-fold) accompanied by depletion of downstream pyrimidine nucleotides was observed, providing strong evidence of robust DHODH target engagement in vivo.

Pathway analysis of differentially abundant metabolites using IPA further highlighted significant alterations in energy metabolism (Fig. S3D). Functional enrichment analysis identified pathways related to cellular growth and proliferation, lipid, nucleotide, and amino acid metabolism, DNA replication and repair, and redox homeostasis as the most significantly affected biological processes (Fig. S3E).

Together, these findings demonstrate that DHODH inhibition induces profound metabolic rewiring in A431 tumors, characterized by accumulation of pyrimidine precursors, disruption of nucleotide homeostasis, and alterations in cellular redox balance.

### Proteomic profiling reveals conserved and cell line-specific molecular responses to DHODH inhibition

**Fig. 5:**
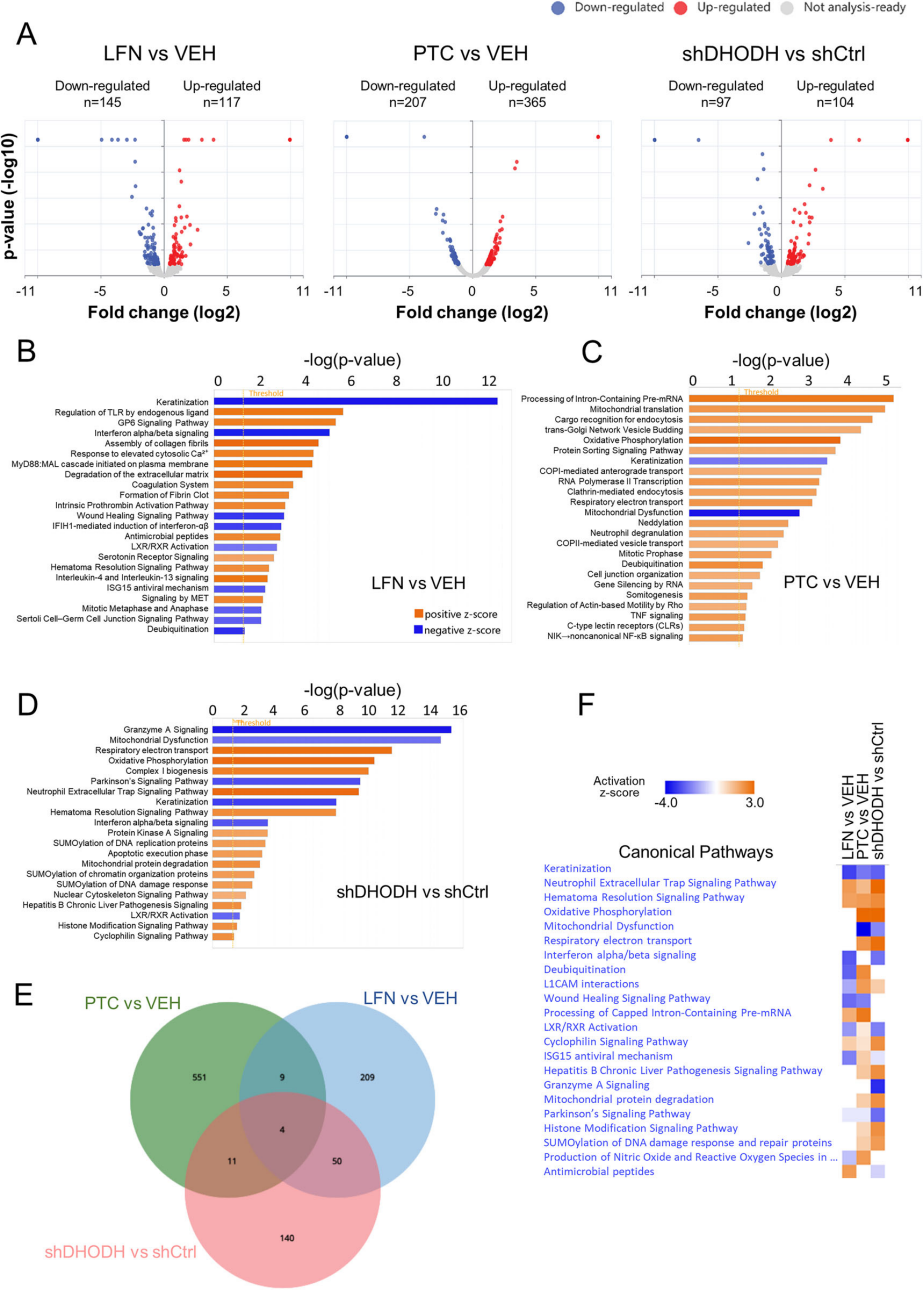
DHODH inhibition induces convergent proteomic alterations in A431 tumors. **A** Label-free quantitative proteomics was performed on A431 xenografts treated with LFN or PTC299 (*n* = 6 per group), as well as on xenografts derived from shDHODH-transduced A431 cells (*n* = 4), and their respective controls (*n* = 6 samples for VEH groups, and 4 xenografts for shCtrl). Volcano plots display differentially expressed proteins (DEPs). Significantly upregulated proteins are shown in red and downregulated in blue (cutoff: |log₂FC| > 1, *p* < 0.05). **B**−**D** Ingenuity Pathway Analysis (IPA®) of DEPs highlighting the most significantly altered canonical pathways (*z*-score >2) following DHODH inhibition in LFN-treated tumors (**B**) PTC-treated tumors (**C**) and shDHODH tumors (**D**). The dashed line indicates the statistical significance threshold (−log(*p*) = 1.3). Orange denotes predicted pathway activation (positive *z*-score) and blue denotes predicted inhibition (negative *z*-score). **E** Venn diagram illustrating the overlap of significantly altered proteins among LFN-, PTC299- and shDHODH-treated tumors. **F** Heatmap showing canonical pathways commonly deregulated across all DHODH-inhibition conditions, with activation scores indicating directionality of pathway regulation (orange = activation; blue = suppression).

**Fig. 6:**
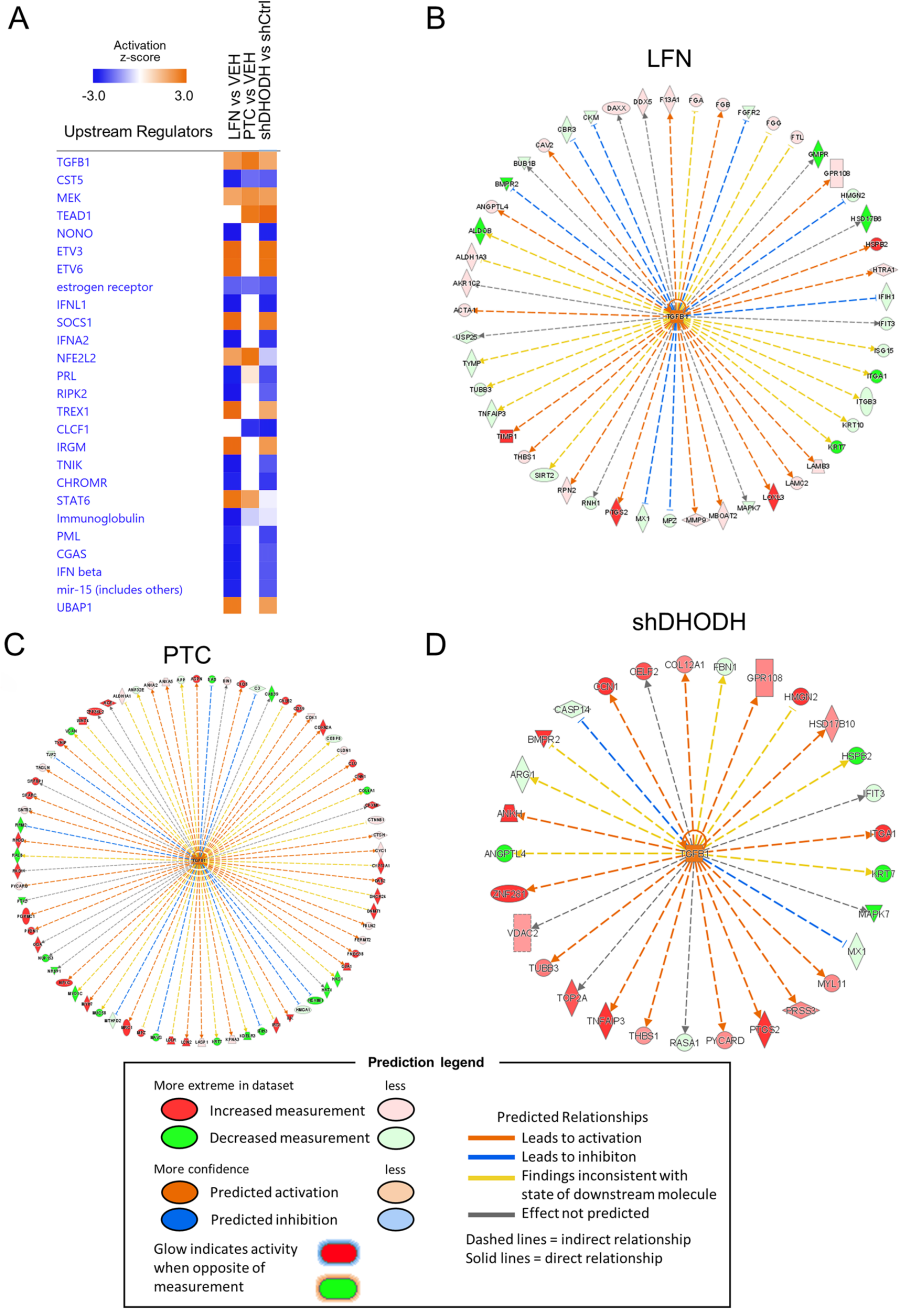
Upstream regulator analysis identifies TGFβ1 and MEK signaling as major drivers of DHODH-inhibition responses. **A** Upstream regulator analysis was performed using QIAGEN Ingenuity® Pathway Analysis (IPA®) on differentially expressed proteins (DEPs) from A431 xenografts treated with LFN, PTC299, or shDHODH compared with their respective controls. Heatmap displays predicted activation states of upstream regulators. Orange indicates predicted activation; blue indicates predicted inhibition. **B**−**D** IPA mechanistic network analyses illustrating predicted activation of TGFβ1 signaling following DHODH inhibition in LFN-treated tumors (**B**) PTC299-treated xenografts (**C**) and shDHODH samples (**D**). The central node represents the inferred upstream regulator, and outer nodes depict downstream targets. Node colors reflect protein expression changes (red = upregulated; green = downregulated). Edge colors and styles indicate predicted regulatory relationships (orange = activation; blue = inhibition; yellow = inconsistent; gray = undetermined; solid lines = direct; dashed lines = indirect).

**Fig. 7:**
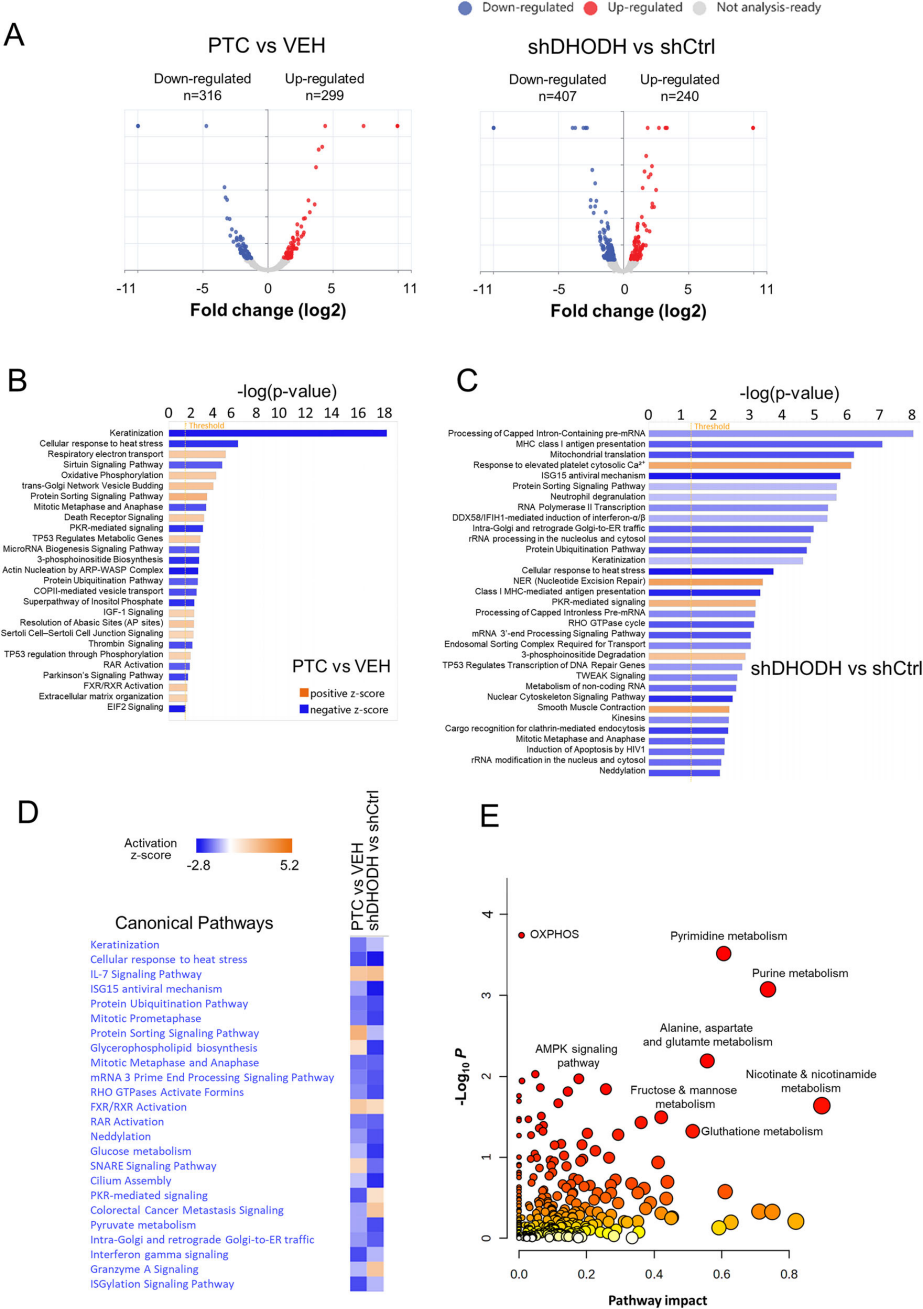
Pharmacological and genetic DHODH inhibition elicits overlapping proteomic signatures in SCC13 tumors. **A** Label-free quantitative proteomics was performed on PTC299-treated SCC13 xenografts (*n* = 6 per group) and on xenografts derived from shDHODH-transduced SCC13 cells (*n* = 4), along with their respective controls (*n* = 6 VEH-treated xenografts, and *n* = 4 shCtrl xenografts). Volcano plots display differentially expressed proteins (DEPs). Significantly upregulated proteins are shown in red and downregulated proteins in blue (cutoff: |log₂FC| > 1, *p* < 0.05). **B**−**C** Ingenuity Pathway Analysis (IPA®) of DEPs highlighting the most significantly affected canonical pathways (*z*-score >2) following DHODH inhibition in PTC-treated xenografts (**B**) and shDHODH tumors (**C**). The dashed line indicates the statistical significance threshold (−log(*p*) = 1.3). Orange indicates predicted pathway activation (positive *z*-score), and blue indicates predicted inhibition (negative *z*-score). **D** Heatmap illustrating canonical pathways commonly altered across both DHODH-inhibition models, with *z*-scores indicating pathway activation (orange) or suppression (blue). **E** Integrated pathway analysis of metabolomic and proteomic datasets derived from xenograft tumors treated with PTC299 or vehicle was performed using the Joint Pathway Analysis module of MetaboAnalyst. Differentially abundant metabolites and proteins were mapped to KEGG pathways and analyzed using topology-based pathway enrichment. The scatter plot displays pathway enrichment significance versus pathway impact, with each circle representing a metabolic pathway. The *x*-axis indicates pathway impact based on topology analysis (degree centrality), while the *y*-axis represents pathway enrichment significance (−log10 *p*). Circle size and color reflect the relative pathway impact and significance, respectively.

To investigate the molecular consequences of DHODH inhibition across distinct cSCC models, label-free quantitative proteomic analyses were performed on A431 and SCC13 xenografts treated with LFN or PTC299, as well as on tumors transduced with shDHODH (Figs. 5–7).

Across all models, DHODH inhibition induced broad proteome remodeling, with PTC299 consistently eliciting the most extensive changes. In A431 tumors, LFN modulated 262 proteins (145 downregulated, 117 upregulated), PTC299 altered 563 (207 downregulated, 356 upregulated), and shDHODH affected 201 (97 downregulated, 104 upregulated) (Fig. 5A). Pathway enrichment analyses revealed that LFN primarily influenced keratinization, extracellular matrix (ECM) remodeling, the interferon regulatory network, and calcium signaling (Fig. 5B). PTC299 prominently altered mitochondrial metabolism, RNA processing, and apoptosis-related pathways (Fig. 5C). shDHODH-mediated DHODH downregulation impacted interferon signaling, mitochondrial metabolism, and keratinization (Fig. 5D).

Comparison of differentially expressed proteins (DEPs) across the three models discerned four commonly altered proteins: keratin II cytoskeletal 6 C (KRT6C), keratin II cytoskeletal 7 (KRT7), annexin A5 (ANXA5), and lipocalin-1 (LCN1). KRT6C and KRT7 are structural components of the epithelial cytoskeleton, and ANXA5 participates in apoptotic cell death. LCN1, an extracellular lipocalin, binds lipophilic molecules, which contributes to cellular metabolism, stress responses, detoxification, and immune signaling (Fig. 5E). Pathway overlap analyses highlighted keratinization as the only pathway consistently affected across all three modes of DHODH inhibition (Fig. 5F).

Subsequently, a predictive upstream regulator analysis was performed to identify regulatory factors whose activation or inhibition could explain the observed proteome alterations. This analysis predicted activation of TGFβ1 and MEK signaling following DHODH inhibition by all three approaches (Fig. 6A, B), consistent with enhanced cell differentiation, metabolic remodeling, and immune responses. A second regulator commonly altered by the three DHODH-inhibiting approaches was CST5, a cysteine proteinase inhibitor, which appeared inhibited. In addition, activation of TEAD1 (a YAP/TAZ-associated transcription factor) and ETV3, along with SOCS1, TREX1, IRGM, several interferon-related transcription factors, and NFE2L2 (NRF2), was predicted. Together, these findings suggest that DHODH inhibition triggers a coordinated network of stress response, redox balance, metabolic adaptation, and differentiation signaling.

Proteomic profiling of SCC13 tumors revealed extensive remodeling upon DHODH inhibition. Compared with their respective control groups, PTC299-treated tumors displayed alterations in 615 proteins (316 downregulated, 299 upregulated), while shDHODH-transduced tumors showed changes in 647 proteins (407 downregulated, 240 upregulated) (Fig. 7A). Pathway enrichment analyses demonstrated that both PTC299 treatment and shRNA-mediated DHODH downregulation elicited pronounced alterations in keratinization, energy metabolism, interferon signaling, and cell division/proliferation pathways, patterns that closely mirrored those observed in A431 tumors following DHODH inhibition (Fig. 7B–D).

Altogether, these regulatory signatures delineate a convergent adaptive response that integrates keratinization, differentiation, metabolic stress, redox balance, and immune modulation downstream of DHODH inhibition in human cSCC cells.

### Integrated metabolomic and proteomic analysis identifies coordinated metabolic pathway remodeling following DHODH inhibition

To obtain a systems-level view of the metabolic consequences of DHODH inhibition, metabolomic and proteomic datasets derived from A431 tumors treated with PTC299 or vehicle were integrated using the joint pathway analysis module of MetaboAnalyst. This approach combines metabolite and protein alterations to identify metabolic pathways that are coordinately dysregulated at multiple molecular levels.

The integrated analysis revealed a significant enrichment of pathways associated with nucleotide metabolism and cellular redox regulation (Fig. 7E). Among the most prominently affected pathways were pyrimidine metabolism, purine metabolism, and alanine, aspartate, and glutamate metabolism, highlighting widespread perturbation of nucleotide biosynthesis and amino acid–linked metabolic flux following DHODH inhibition. In addition, pathways involved in nicotinate and nicotinamide metabolism, glutathione metabolism, and oxidative phosphorylation (OXPHOS) were significantly enriched, suggesting alterations in mitochondrial function and cellular redox homeostasis.

Notably, pyrimidine metabolism displayed the highest pathway impact score, consistent with the direct inhibition of DHODH and the accumulation of upstream pyrimidine intermediates observed in the metabolomic dataset. Purine metabolism was also significantly enriched, indicating compensatory remodeling of nucleotide pools in response to impaired pyrimidine biosynthesis. Furthermore, the enrichment of glutathione metabolism and nicotinate/nicotinamide metabolism suggests adaptive responses to oxidative and metabolic stress induced by DHODH inhibition.

Together, these integrated analyses demonstrate that DHODH inhibition triggers coordinated metabolic reprogramming affecting nucleotide metabolism, amino acid metabolism, mitochondrial function, and cellular redox balance.

### A shift toward a more differentiated state underlies the antitumor effect of DHODH inhibition in A431 and SCC13 tumors

**Fig. 8:**
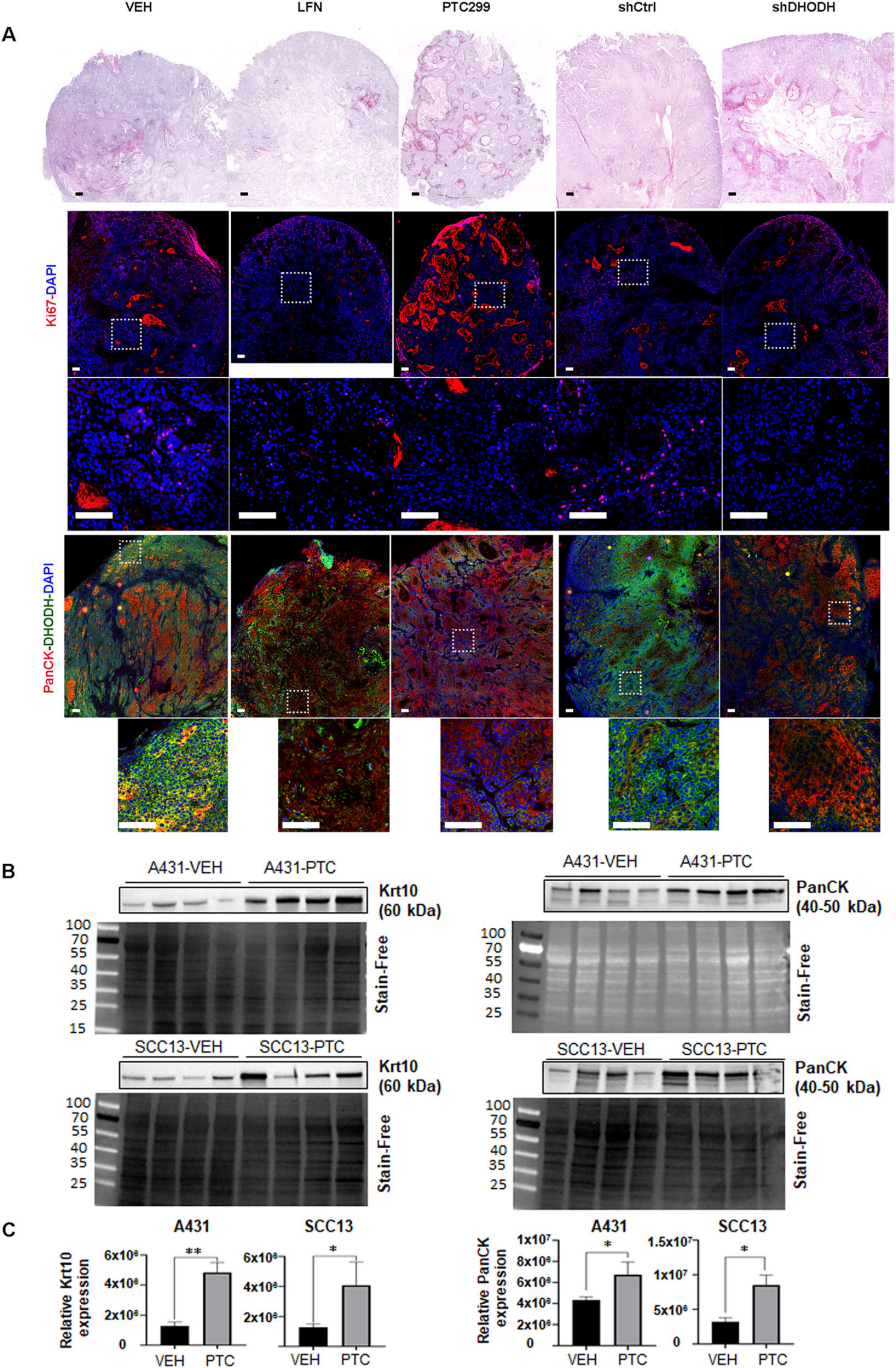
DHODH inhibition promotes keratinocyte differentiation in A431 and SCC13 xenografts, with PTC299 demonstrating the most pronounced effect. **A** Representative image of A431 tumor sections stained with H&E (top), immunostained for Ki67 (red) and DAPI (blue) (middle), and co-stained for PanCK (red), DHODH (green), and DAPI (blue) (bottom). Treatment groups include VEH, LFN, PTC299, shCtrl, and shDHODH. Insets show magnified regions from boxed regions. Scale bars = 150 µm. **B** Western blot analysis of keratin 10 (Krt10, left) and PanCK (right) in A431 and SCC13 xenografts treated with PTC299 or vehicle. Stain-free gels show the total protein as a loading control. **C** Densitometric quantification of western blot signals from (**B**) normalized to total protein (Stain-Free). Data represent mean ± SEM (*n* = 4 tumors per group). Statistical significance was assessed using an unpaired two-tailed Student’s *t* test (*p < 0.05, **p < 0.01).

To validate the proteomic evidence suggesting altered keratinization and modification in cell proliferation following DHODH inhibition, we first examined the histology of xenografts, which revealed clear morphological differences among treatment groups (Fig. 8A). In VEH-treated xenografts and shCtrl groups, the tumors displayed dense cellularity with extensive viable tumor regions and minimal keratinization, consistent with an actively proliferating phenotype. In LFN-treated xenografts, reduced cellular density and increased necrotic areas were observed. In PTC299-treated xenografts and the shDHODH group, the tumor architecture exhibits pronounced keratinized regions, cystic or keratin pearl–like structures, and reduced viable tumor tissue, indicative of enhanced differentiation and possible tumor regression (Fig. 8A). To provide a quantitative evaluation of these phenotypes, we then examined the expression of ki67 and Pan-cytokeratin (PanCK) in A431 xenografts. Ki67 immunostaining revealed abundant proliferating cells throughout the tumors in vehicle-treated and shCtrl xenografts. In contrast, Ki67-positive cells were largely absent from the central regions of LFN- and PTC299-treated tumors, as well as in shDHODH xenografts, where labeling was mainly restricted to the tumor periphery (Fig. 8A). PanCK immunostaining showed an increased number of cells with strong PanCK expression in all DHODH-inhibited conditions (Fig. 8A). DHODH immunostaining confirmed efficient knockdown in shDHODH xenografts and demonstrated reduced DHODH protein levels in LFN- and PTC299-treated tumors (Fig. 8A), suggesting the presence of a feedback mechanism that downregulates DHODH expression upon inhibition of its enzymatic activity.

To further quantify these observations, Western blot analyses were performed. Results demonstrated a pronounced increase in Keratin 10 (Krt10) and PanCK expression in LFN- and PTC299-treated A431 and SCC13 xenografts compared with their respective vehicle-treated controls (Fig. 8B). Densitometric analysis confirmed significant upregulation of Krt10 and PanCK levels in PTC299-treated A431 and SCC13 xenografts (Fig. 8C), consistent with a more differentiated and less proliferative tumor phenotype driven by DHODH inhibition. Western blot analysis of DHODH protein expression in A431 and SCC13 xenografts treated with PTC299 corroborated the immunostaining results, demonstrating reduced DHODH levels in both models compared with their vehicle-treated counterparts (Fig. S4).

Overall, the histopathological features and immunostaining findings are consistent with the molecular data, indicating that DHODH inhibition promotes a differentiated tumor phenotype and markedly suppresses proliferation compared with control groups. Among the inhibitors tested, PTC299 induces a stronger differentiation response and a more pronounced reduction in tumor proliferation than LFN.

## Discussion

In this study, we demonstrate that inhibition of DHODH, either pharmacologically (with LFN or PTC299) or through genetic silencing (shDHODH), profoundly suppresses tumor growth and remodels metabolic, proteomic, redox, and differentiation signatures in cSCC xenograft models (A431 and SCC13). All three approaches reduced tumor progression, with PTC299 producing the most significant reduction in tumor mass. Collectively, these data identify DHODH as a central metabolic hub that links de novo pyrimidine synthesis, mitochondrial bioenergetics, redox homeostasis, and cellular differentiation in cSCC.

Beyond cSCC, elevated DHODH expression has been associated with enhanced proliferation, metastatic potential, and poor prognosis in diverse malignancies, including lung, breast, colorectal cancers, and neuroblastoma [11, 21, 23–25], underscoring the broad therapeutic relevance of DHODH across cancer types. DHODH occupies a unique position as the only mitochondrial enzyme in the de novo pyrimidine biosynthesis pathway, coupling dihydroorotate oxidation to ubiquinone reduction in the respiratory chain. Studies have demonstrated that mitochondrial respiration is required to sustain DHODH activity and thereby support de novo pyrimidine synthesis and cancer cell proliferation, even in highly glycolytic tumors. This dependency has been demonstrated in breast cancer and melanoma mouse models and later extended to glioblastoma [26, 27]. Furthermore, DHODH activity has been shown to be critical for efficient early tumor initiation [28]. Consequently, DHODH inhibition simultaneously compromises nucleotide supply and mitochondrial function, leading to cell cycle arrest, differentiation, or apoptosis [19]. This complex mechanism has been documented in proliferative cancers relying on high nucleotide demand, such as acute myeloid leukemia (AML), where DHODH inhibition induces differentiation [29], and melanoma, where it arrests proliferation [30].

Despite its promise, therapeutic exploitation of DHODH inhibition has been limited by several challenges. LFN, an FDA-approved DHODH inhibitor used for autoimmune disorders, exhibits only modest antitumor efficacy in preclinical cancer models, likely due to suboptimal potency, partial enzyme blockade, and heterogeneous metabolic dependence [31, 32]. Indeed, a growing body of evidence demonstrates that tumors are not metabolically uniform, displaying metabolic dependencies and niche adaptations both between patients (intertumoral) and within individual lesions (intratumoral) [33, 34]. In epithelial cancers, single-cell and spatial profiling studies have revealed diverse keratinocyte sub-populations with different gene expression profiles [35]. Thus, it is possible to have distinct metabolic niches characterized by differential reliance on de novo pyrimidine synthesis, fatty acid oxidation, or salvage pathways even within a single tumor. Consequently, certain regions within a tumor may be highly DHODH-dependent, while others bypass DHODH inhibition via salvage pathways or alternative energy routes. Recent proteomic-based metabolic scoring of patient-derived tumors revealed that LFN preferentially inhibits cSCC tumors with low metabolic scores, suggesting that tumor metabolic profile strongly influences therapeutic sensitivity [5]. The heterogeneous metabolic landscape of tumors may explain the limited efficacy with DHODH-targeting drugs in clinical trials for tumor therapy [20]. This observation underscores the importance of personalized metabolic characterization in predicting therapeutic response and highlights the limitation of DHODH inhibitors as a “one-size-fits-all” strategy for cancer treatment [5].

Our metabolomic and proteomic analyses revealed that both pharmacological and genetic inhibition of DHODH lead to profound metabolic perturbations, including accumulation of dihydroorotic acid, alterations in aspartate and pyruvate metabolism, and changes in redox-related metabolites such as ascorbate and reduced glutathione. These alterations indicate a dual disruption of nucleotide biosynthesis and cellular redox homeostasis, two metabolic processes that are tightly linked to proliferative capacity. Consistent with this metabolic phenotype, tumors treated with DHODH inhibitors exhibited increased expression of keratinocyte differentiation markers (Krt10, PanCK), suggesting a shift toward a more differentiated and less proliferative cellular state. In parallel, cell-cycle analyses revealed a marked accumulation of cells in S phase, indicative of replication stress. In agreement, it has been recently shown that nucleotide limitation can promote replication stress–mediated differentiation, thereby limiting tumor cell proliferation [36].

Pathway enrichment analyses further confirmed dysregulation of pyrimidine metabolism and glutathione pathways, consistent with a “metabolic catastrophe” driven by pyrimidine depletion and oxidative stress [19]. These findings parallel those in AML, where nucleotide depletion induces differentiation [29], supporting a conserved mechanism by which nucleotide scarcity and redox imbalance jointly drive differentiation in epithelial tumors. Although both A431 and SCC13 ultimately respond to DHODH inhibition, consistent with the similar metabolic and proteomic rewiring observed across models, the inclusion of a cell line with primary resistance to DHODH inhibitors would indeed provide valuable insight into determinants of intrinsic sensitivity and adaptive responses. In addition, a complete genetic ablation of DHODH followed by rescue experiments would offer a complementary mechanistic framework to further delineate the specific contribution of DHODH activity to these phenotypes.

From a therapeutic perspective, the superior efficacy of PTC299 in our models likely reflects its greater potency and ability to inhibit DHODH in metabolically active tumor subsets. In contrast, LFN with a weaker ability of inhibition may be less effective in tumors with high metabolic plasticity. These observations emphasize the importance of metabolic profiling and biomarker-driven patient selection to guide DHODH-targeted therapy. Looking forward, identifying predictive biomarkers of DHODH dependency in cSCC, such as DHODH expression level, metabolic score, or salvage enzyme activity, will be critical for precision treatment. Our study identified a molecular signature associated with three different modalities of DHODH inhibition, which could be used for clinical studies. Moreover, combination therapies may further enhance the DHODH-inhibition strategy efficacy. Notably, our proteomic and metabolomics data suggest that adaptive resistance to DHODH inhibition may arise via metabolic rerouting, including increased salvage nucleotide uptake, reprogramming of energy metabolism, and redox buffering. Rational combination approaches that simultaneously block these compensatory pathways may therefore maximize therapeutic impact. For instance, co-targeting DHODH with immune checkpoint inhibitors, ferroptosis inducers, or nucleotide salvage inhibitors could overcome adaptive resistance [37, 38]. Our findings also indicate alteration of protein synthesis mechanisms in response to DHODH inhibition, which might suggest additional co-targeting approaches.

Finally, Pharmacologic inhibition of DHODH reduces intracellular pyrimidine pools, thereby impairing DNA and RNA synthesis. Because activated T and B lymphocytes rely heavily on de novo pyrimidine synthesis, rather than salvage pathways, DHODH inhibition selectively suppresses clonal expansion and effector function of these immune cells [39, 40]. This mechanism underlies the established clinical use of DHODH inhibitors in autoimmune diseases such as Rheumatoid arthritis and Multiple sclerosis [41]. This well-established immunomodulatory activity raises an important consideration in oncology, as inhibition of DHODH could theoretically impair adaptive antitumor immune responses, particularly in the context of immunotherapy. At the same time, emerging evidence suggests that DHODH inhibition may, under certain conditions, enhance antitumor immunity. A recent study demonstrated that CRISPR-mediated deletion of DHODH in tumor cells inhibited tumor growth in immunocompetent mice but not in immunodeficient mice [38]. Importantly, DHODH inactivation enhanced infiltration of IFNγ-secreting CD8⁺ T cells and improved the efficacy of PD-1 blockade in syngeneic tumor models [38]. These findings underscore the context-dependent nature of DHODH inhibition in cancer. While systemic DHODH blockade may exert immunosuppressive effects on proliferating lymphocytes, tumor-intrinsic DHODH inhibition may increase tumor immunogenicity and susceptibility to immune-mediated killing. Our xenograft studies, performed in immunodeficient mice, do not capture these immune-dependent mechanisms. Therefore, future studies in immunocompetent systems will be critical to delineate the balance between potential suppression of adaptive immunity and enhancement of tumor cell vulnerability to cytotoxic T cells, particularly in combination with immune checkpoint inhibitors.

## Conclusion

Our integrated proteomic and metabolomics investigation demonstrates that DHODH inhibition in cSCC not only suppresses tumor growth but also triggers metabolic reprogramming, redox adaptation, and cellular differentiation. While LFN provides proof of principle, PTC299 emerges as a more potent and mechanistically robust inhibitor. These findings establish DHODH as a promising therapeutic target in cSCC and highlight the need for biomarker- and metabolism-based patient stratification to fully exploit this metabolic vulnerability.

## Supplementary information

Appendix S1- Additional supplementary material file

Supplementary figure legends

Fig. S1

Fig. S2

Fig. S3

Fig. S4

full length western blots used in the manuscript

## Data Availability

The authors declare that the data supporting the findings of this study are available within the paper and its Supplementary Information files. The proteomics data have been deposited to the ProteomeXchange Consortium via the PRIDE partner repository with the dataset identifier PXD070860. Additional data are available from the corresponding author upon reasonable request.
