# Supplementary material for: DHODH inhibition suppresses cutaneous squamous cell carcinoma growth by the induction of differentiation through perturbation of the cellular redox balance
Source: Cell Death Dis. 2026 Apr 28;17(1):566. doi: 10.1038/s41419-026-08815-w (PMC13260998; doi:10.1038/s41419-026-08815-w)
Supplement: Supplementary file 1 — Appendix S1- Additional supplementary material file [file 41419_2026_8815_MOESM1_ESM.docx]

**Additional supplementary material file**

**Measurement of cell growth (SRB assay)**

Cell viability was assessed using the sulforhodamine B (SRB) assay. Briefly, cells were seeded in 96-well plates at a density of 5 × 10³ cells/well and allowed to adhere overnight. The following day, cells were treated with LFN or PTC299 at various concentrations for 24 and 48 hours.

After treatment, cells were fixed by the addition of cold 10% (w/v) trichloroacetic acid (TCA) and incubated at 4°C for at least 1 hour. Plates were then washed with distilled water five times to remove excess TCA and air-dried. Fixed cells were stained with 0.4% (w/v) SRB in 1% acetic acid for 30 minutes at room temperature. Unbound dye was removed by washing the plates with 1% acetic acid five times, followed by air drying.

The bound dye was solubilized with 10 mM Tris base, and the absorbance was measured at 510 nm using CLARIOstar Plus microplate reader. The percentage of cell viability was calculated relative to untreated control wells.

**Lentiviral vectors and cell transduction**

Lentiviral vectors expressing shRNA against DHODH were purchased from Sigma-Aldrich (TRCN0000025839). Cells were transduced with lentiviral vectors (pLKO U6-DHODH 868-hPGK-Puro for shDHODH; pLKO U6-shCtrl-hPGK-Puro for shPLKO control). Briefly, 100000 cells were seeded in a 6-well plate. The next day, cells were incubated with lentiviral particles for 24 hours. Three days after transduction, cells were selected with puromycin. Specific inhibition of human DHODH protein expression was verified by western blot before transplantation into mice.

**Western blot**

***Protein Extraction:*** Cells were lysed in ice-cold RIPA lysis buffer (Thermo Fischer scientific) supplemented with protease and phosphatase inhibitor cocktail (Halt™ Protease and Phosphatase Inhibitor Cocktail, Thermo Fisher Scientific). Cell lysates were incubated on ice for 20 minutes and then centrifuged at 13000 rpm for 20 minutes at 4°C. The supernatant was collected, and protein concentration was determined using the BCA protein assay kit (Thermo Fisher Scientific).

For xenograft samples, sections of the xenograft were put in a hypotonic buffer (2.5 mM Tris/HCl, pH 7.5 and 2.5 mM MgCl2) supplemented with protease and phosphatase inhibitor cocktail (Halt™ Protease and Phosphatase Inhibitor Cocktail, Thermo Fisher Scientific) and grinded with Precellys® Evolution. The tubes were than centrifuge at 13 000 rpm for 5 mins at 4°C. The supernatant was collected, adding 10x RIPA analogue (500 mM Tris-HCl pH 7.4, 1.5 M NaCl, 10% Triton X-100, 1% SDS, and 5% sodium deoxycholate), incubated for 20 min on ice and then centrifuged at 13000 rpm for 20 minutes at 4°C. The supernatant was collected, and protein concentration was determined using the BCA protein assay kit (Thermo Fisher Scientific).

***SDS-PAGE and Transfer:*** Equal amounts of protein (30 µg) were mixed with 6x Laemmli sample buffer, heated at 95°C for 5 minutes, and loaded onto precast SDS-PAGE gels (4–20% Mini-PROTEAN® TGX™ Stain Free Precast Gels, Bio-Rad). Electrophoresis was performed at 55–120 V until appropriate separation was achieved. Proteins were transferred onto nitrocellulose membranes (Amersham™ Protran® Western blotting membranes, nitrocellulose, Merck) using eBlot™ L1 Protein Transfer System (GenScrip).

***Blocking and Antibody Incubation:*** Membranes were blocked with 2% ECL prime blocking reagent (CYTIVA) for 1 hour at room temperature. After blocking, membranes were then incubated overnight at 4 °C with a 1:1000 dilution of the anti-DHODH (ABC138, Merck), anti-PanCK (sc-81714, Santa-Cruz), anti-Krt10 (ab76318, abcam) diluted in the blocking buffer. The following day, membranes were washed with TBST (3 × 10 minutes) and incubated with a 1:10000 dilution of an anti-immunoglobulin horseradish peroxidase-linked antibody (Eurobio scientific) for 1 h. After additional washes, protein bands were detected using enhanced chemiluminescence (ECL) reagent (Biorad), and visualized using iBright Imaging System (Thermo Fisher Scientific).

***Data Analysis*:** Band intensities were quantified using iBright analysis software. Target protein expression levels were normalized to housekeeping proteins such as β-actin or to total lane protein. Data were analyzed from at least three independent experiments.

**Proteome analyses by mass spectrometry**

***Sample preparation and protein digestion:*** Protein samples were solubilized in Laemmli buffer and separated by SDS–PAGE. After colloidal blue staining, each lane was excised and further cut into approximately 1 × 1 mm gel pieces. The gel pieces were destained in 25 mM ammonium bicarbonate containing 50% acetonitrile (ACN), rinsed twice with ultrapure water, and dehydrated in ACN for 10 min. After removal of ACN, the gel pieces were air-dried at room temperature, covered with sequencing-grade trypsin solution (10 ng/µL in 50 mM ammonium bicarbonate), rehydrated at 4 °C for 10 min, and incubated overnight at 37 °C.

Peptides were first extracted by incubating the gel pieces for 15 min in 50 mM ammonium bicarbonate with gentle shaking. The supernatant was collected, and extraction was repeated twice with a H₂O/ACN/formic acid (47.5:47.5:5, v/v/v) solution for 15 min each. The combined extracts were pooled and dried in a vacuum centrifuge. The resulting peptide digests were resuspended in 0.1% formic acid prior to LC–MS/MS analysis.

***nLC-MS/MS analysis and Label-Free Quantitative Data Analysis:*** Peptide mixtures were analyzed using an Ultimate 3000 nanoLC system (Dionex, Amsterdam, The Netherlands) coupled to an Orbitrap Fusion™ Lumos™ Tribrid™ Mass Spectrometer (Thermo Fisher Scientific, San Jose, CA).

Ten microliters of each peptide digest were loaded onto a C18 PepMap™ trap column (300 µm × 5 mm, LC Packings) at a flow rate of 10 µL/min. Peptides were separated on a C18 PepMap™ analytical column (75 µm × 50 cm, LC Packings) using a linear gradient of 4–25% solvent B over 87 min, followed by an increase from 25% to 40% solvent B in 30 min (solvent A: 0.1% formic acid in water; solvent B: 0.1% formic acid in 80% ACN). The flow rate was set to 200 nL/min.

The mass spectrometer was operated in positive ion mode with a spray voltage of 2 kV. Data were acquired in data-independent acquisition (DIA) mode using Xcalibur 4.4 software. Each cycle consisted of a full MS scan (resolution 60,000; AGC target 4 × 10^5; maximum injection time 50 ms; scan range 350–1400 m/z) followed by 34 DIA MS/MS scans (resolution 30,000; AGC target 5 × 10^5; maximum injection time 54 ms) with variable-width isolation windows covering 380–980 m/z. Higher-energy collisional dissociation (HCD) was performed with a collision energy of 25%.

***Database search and results processing:*** DIA data were analyzed with Chimerys in Proteome Discoverer 3.1 (Thermo Fisher Scientific) against the *Homo sapiens* protein database (2021-03 release, 20,324 entries). Spectra corresponding to peptides <350 Da or >5000 Da were excluded. The maximum precursor mass tolerance was set at 10 ppm. Up to two missed cleavages were allowed. Carbamidomethylation of cysteines (+57 Da) was set as a fixed modification, while oxidation of methionine (+16 Da) was considered as a variable modification. Only peptides with high confidence (corresponding to a 1% false discovery rate at the peptide level) were retained ^1^. Quantification was performed using the Fragment Ion Quantifier node, and normalization was based on the total human peptide abundance. Protein ratios were calculated as the median of all pairwise peptide ratios. Statistical analysis was performed using a t-test with Benjamini–Hochberg correction. Only proteins quantified with at least two peptides and with an adjusted *P* < 0.05 were considered significant. The mass spectrometry proteomics data have been deposited to the ProteomeXchange Consortium via the PRIDE ^2^ partner repository with the dataset identifier PXD070860.

**Semi-targeted Metabolomics**

Metabolites were extracted from tumors following the EtOH/Hepes protocol described in ^3^. Briefly, pre-weighed, frozen tumor samples were homogenized in 1 ml of EtOH/Hepes (8:2 v/v, 1 mM, pH 7) containing internal standards: Succinic acid-2,2,3,3-d_4_, L-Aspartic acid-^13^C_4_,^15^N, L-Tyrosine-^13^C_9_ and Adenosine-d_14_ 5′-triphosphate. Tissue disruption was performed using a TissueLyzer (Qiagen) with 3 mm stainless steel beads (2 cycles of 30 Hz for 2 min at 4°C). Lysates were heated at 80°C for 3 min and subsequently evaporated using a rotary evaporator. The resulting residue was reconstituted in MilliQ water (10 μL/mg tissue), insoluble material was removed by centrifugation (1 h, 4°C, 21,000 × g), and the supernatant was subjected to ultrafiltration using a Nanosep 10K Omega cartridge (Pall) for 15 min at 4°C and 14,000 × g. Metabolites were separated via liquid chromatography using either high-performance ion chromatography (HPIC; ICS6000, Thermo Electron) or high-performance liquid chromatography (HPLC; Vanquish Flex, Thermo Electron), with AS11-HC-4 µm (250 × 2 mm) and Acclaim RSLC PAII (2.2 µm, 120 Å, 2.1 × 150 mm) analytical columns, respectively. HPIC separations were conducted at a flow rate of 0.38 mL/min with the discontinuous KOH gradient as previously described ^3^. For HPLC, metabolites were separated at 0.25 mL/min using the mobile phases (A) 50 mM formic acid pH 3 (adjusted with ammonia) and (B) methanol. The gradient was 0% B for 0.5 min, increased to 7% over 5 min, rapidly raised to 40% in 0.5 min, maintained for 3 min, then increased to 90% over 1 min, held for 5 min, and returned to 0% in 1 min, followed by a 10-min column re-equilibration before subsequent injections. For both chromatographic methods, metabolite detection was performed using a high-resolution Orbitrap mass spectrometer (Exploris 120, Thermo Fisher Scientific) with an EASY-IC ion source operating in negative (HPIC and HPLC) and positive (HPLC) ion mode with scan-to-scan lock-mass correction. Data acquisition was conducted using Xcalibur 4.7 software (Thermo Fisher Scientific). A total of 168 targeted metabolites were quantified using TraceFinder 5.2 SP3 software (Thermo Fisher Scientific) from full MS scans (m/z 70-1,000) acquired at a resolution of 60,000 with data-dependent MS² scans acquired at 15,000 resolution with an HCD collision energy at 30%. Metabolite identification was based on retention time, accurate mass, natural isotopic distribution and MS² fragment patterns.

**Integrated metabolomic and proteomic pathway analysis**

Integrated pathway analysis of metabolomic and proteomic datasets was performed using the Joint Pathway Analysis module of MetaboAnalyst (version 5.0). Differentially abundant metabolites identified by semi-targeted metabolomic profiling and significantly regulated proteins identified by quantitative proteomics were used as input for the analysis.

Metabolites were annotated using HMDB identifiers, and proteins were mapped to their corresponding gene symbols. Integrated pathway enrichment analysis was performed using the path_viewer_v1.8 algorithm, which combines metabolite and protein information within curated metabolic pathways from the KEGG database. Pathway topology analysis was conducted using degree centrality to assess the relative importance of altered nodes within each pathway network.

For the integration parameters, tight integration by combining queries was selected, and equal weighting was applied to metabolite and protein datasets. Enrichment analysis was performed using the hypergeometric test, and pathway impact scores were calculated based on topology analysis. Pathways were ranked according to their enrichment significance and impact scores, and results were visualized as a pathway impact scatter plot generated by MetaboAnalyst.

**Cell-cycle analysis by PI and EdU incorporation**

Cell-cycle distribution was assessed using the Click-iT EDU FLOW Cytometry Assay Kit (Thermo Fisher), according to the manufacturer's instructions. Briefly, cells were incubated with EdU (10 μM) for 4 hours prior to harvesting to label cells undergoing active DNA synthesis. After harvesting cells with trypsin and washing with PBS, cells were fixed and permeabilized. Following fixation, incorporated EdU was detected using the Click-iT reaction according to the manufacturer's instructions. Cells were then treated with RNase A (100 μg/mL) and stained with propidium iodide (PI, 50 μg/mL) to quantify total DNA content. Samples were analyzed using a BD flow cytometer (BD Biosciences). At least 10,000 events per sample were acquired. Cell-cycle distribution (G0/G1, S, and G2/M phases) was determined based on DNA content and EdU incorporation using appropriate gating strategies.

**Immunofluorescence and H&E staining**

Tumors were harvested, fixed in formalin for 24 hours at room temperature, and embedded in paraffin. Sections of 4 μm thickness were cut using a rotary microtome and mounted onto Superfrost Plus microscope slides. For Hematoxylin and Eosin (H&E) staining, slides were deparaffinized in toluene, rehydrated through a graded ethanol series, and stained with hematoxylin followed by eosin according to standard protocols. For immunofluorescence, slides were deparaffinized in xylene and rehydrated, followed by antigen retrieval in citrate buffer (10 mM, pH 6.0) using a pressure cooker. After blocking with 5% BSA in PBS for 1 hour at room temperature, sections were incubated overnight at 4°C with primary antibodies diluted in blocking solution. The next day, slides were incubated with appropriate fluorophore-conjugated secondary antibodies for 1 hour at room temperature, counterstained with DAPI, and mounted using an anti-fade mounting medium. Images were acquired using a fluorescence microscope (ECHO Revolution).

**Statistical analysis**

All data are presented as mean ± SEM from at least three independent experiments. Statistical analyses were performed using GraphPad Prism software. Data normality was assessed using the Kolmogorov–Smirnov test. Comparisons between two groups were evaluated using an unpaired two-tailed Student’s t-test. For in vivo tumor growth curves, statistical differences between groups over time were analyzed using two-way ANOVA followed by Sidak’s multiple comparison test. Final tumor weights and other single-time-point measurements were analyzed by one-way ANOVA or t-test as appropriate. *P*-values < 0.05 (*), < 0.01 (**), <0.001 (***) and < 0.0001 (****) were considered significant.

**References**

1 Käll L, Canterbury JD, Weston J, Noble WS, MacCoss MJ. Semi-supervised learning for peptide identification from shotgun proteomics datasets. *Nat Methods* 2007; **4**: 923–925.

2 Deutsch EW, Bandeira N, Perez-Riverol Y, Sharma V, Carver JJ, Mendoza L *et al.* The ProteomeXchange consortium at 10 years: 2023 update. *Nucleic Acids Res* 2023; **51**: D1539–D1548.

3 Pinson B, Moenner M, Saint-Marc C, Granger-Farbos A, Daignan-Fornier B. On-demand utilization of phosphoribosyl pyrophosphate by downstream anabolic pathways. *J Biol Chem* 2023; **299**: 105011.
