# Supplementary material for: DHODH inhibition suppresses cutaneous squamous cell carcinoma growth by the induction of differentiation through perturbation of the cellular redox balance
Source: Cell Death Dis. 2026 Apr 28;17(1):566. doi: 10.1038/s41419-026-08815-w (PMC13260998; doi:10.1038/s41419-026-08815-w)
Supplement: Supplementary file 2 — Supplementary figure legends [file 41419_2026_8815_MOESM2_ESM.docx]

**Supplementary Figures and figure legends**

**Figure S1. DHODH inhibition is well tolerated and did not induce weight loss in tumor-bearing mice.**

**(A-B)** NSG mice bearing A431 (**A**) and SCC13 (**B**) xenografts were treated orally with leflunomide (LFN), PTC299, or carboxymethylcellulose (vehicle, veh) once tumors reached the desired size. Body weight was monitored at the indicated time points. Data represent mean ± SEM. Both LFN and PTC299 were well tolerated, with no significant weight loss observed and overall body weight remaining stable during the treatment period.

**Figure S2. DHODH inhibition induces S-phase arrest in tumor cells.**

Cell-cycle distribution was analyzed by flow cytometry following propidium iodide (PI) staining and EdU incorporation. A431 cells were treated with PTC299 or vehicle (VEH), and the percentages of cells in G0/G1, S, and G2/M phases were quantified. Data represent mean ± SEM from three independent experiments. Statistical significance was determined using two-way repeated-measures ANOVA followed by Bonferroni’s multiple comparisons test (***P* < 0.01, ****P* < 0.001; ns, not significant).

**Figure S3. PTC299-mediated DHODH inhibition triggers pyrimidine precursor accumulation, metabolic rerouting, and oxidative stress responses**

Semi-targeted metabolomic profiling was performed on A431 xenografts treated with PTC299 or VEH (n=5 per group). (**A**) Heatmap illustrating differential metabolite abundances across conditions. Colors represent low (red) and high (green) metabolite levels.

(**B**) Volcano plots show the differentially expressed metabolites in PTC299-treated vs VEH-treated xenografts. A total of 159 metabolites were identified.

(**C**) Relative abundances of dihydroorotic acid, UTP, CTP, aspartic acid, and reduced glutathione in PTC299-treated vs. vehicle xenografts. Data represent mean ± SD for n = 5 biological replicates. Statistical significance was assessed by multiple t-tests with p < 0.05 and FDR 1% (**P* < 0.05, ***P* < 0.01).

(**D**) Ingenuity Pathway Analysis (IPA®) of differentially abundant metabolites highlighting the top significantly affected canonical pathways (z-score > 2). The dashed line denotes the significance threshold (−log(*P*) = 1.3).

(**E**) Gene Ontology enrichment analysis of the differentially expressed metabolites shows significantly enriched molecular and cellular functions. Only GO terms that were significantly overrepresented (*P* < 0.05) are shown.

**Figure S4. PTC299 reduces DHODH protein levels in A431 and SCC13 xenografts.**

**(A–B) Western blot analysis of DHODH protein expression in A431 (A) and SCC13 (B) xenografts treated with vehicle (VEH) or PTC299 (PTC). Stain-Free total protein is shown as a loading control. Bar graphs display densitometric quantification of DHODH expression normalized to total protein. Data are shown as mean ± SEM (n = 4 tumors per group). Statistical significance was calculated using an unpaired two-tailed Student’s t-test (**P* < 0.05; ***P* < 0.01).**
