# Supplementary figures and images for: DHODH inhibition suppresses cutaneous squamous cell carcinoma growth by the induction of differentiation through perturbation of the cellular redox balance
Source: Cell Death Dis. 2026 Apr 28;17(1):566. doi: 10.1038/s41419-026-08815-w (PMC13260998; doi:10.1038/s41419-026-08815-w)

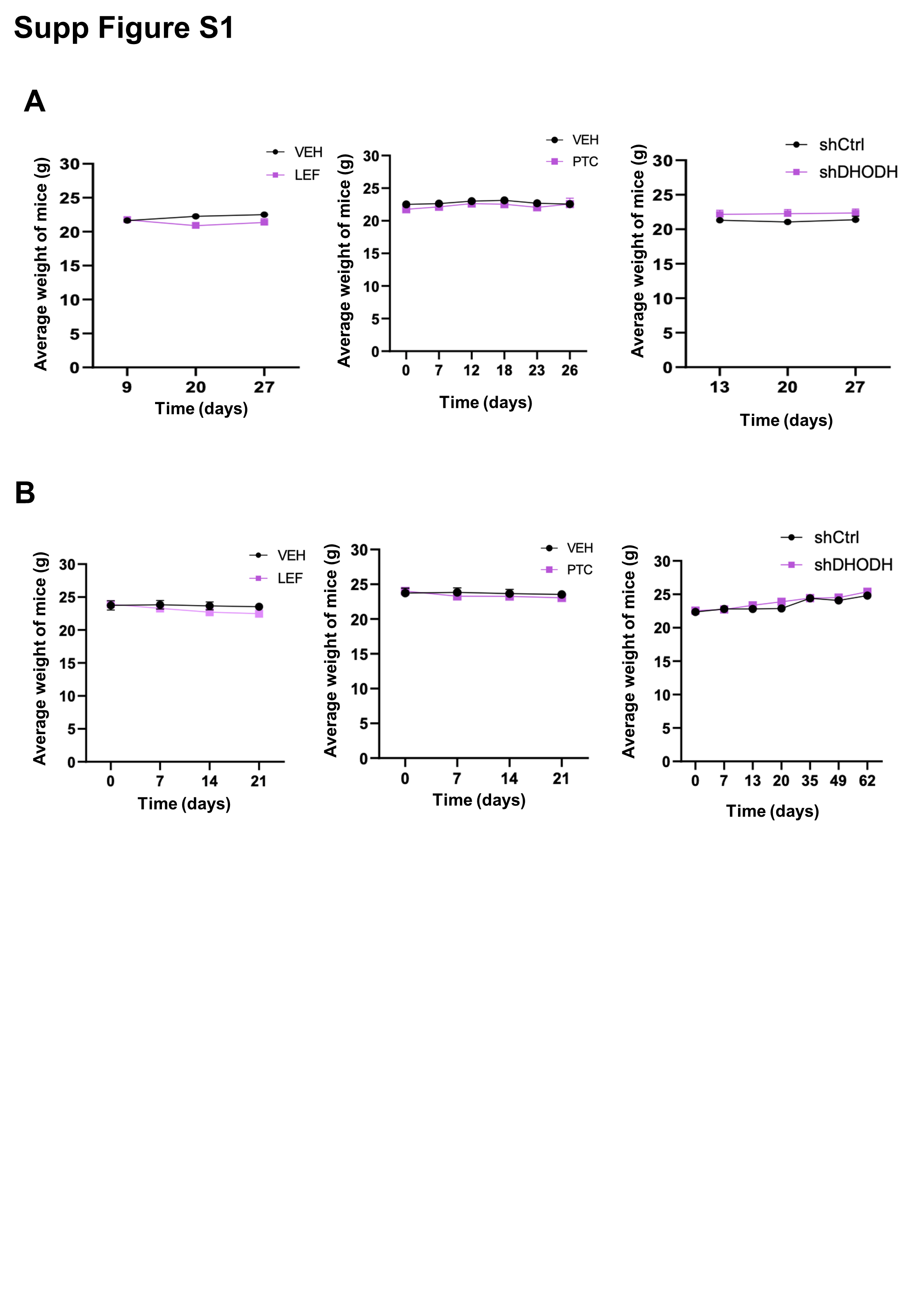

Supplement: Supplementary file 3 — Fig. S1 [file 41419_2026_8815_MOESM3_ESM.tif]

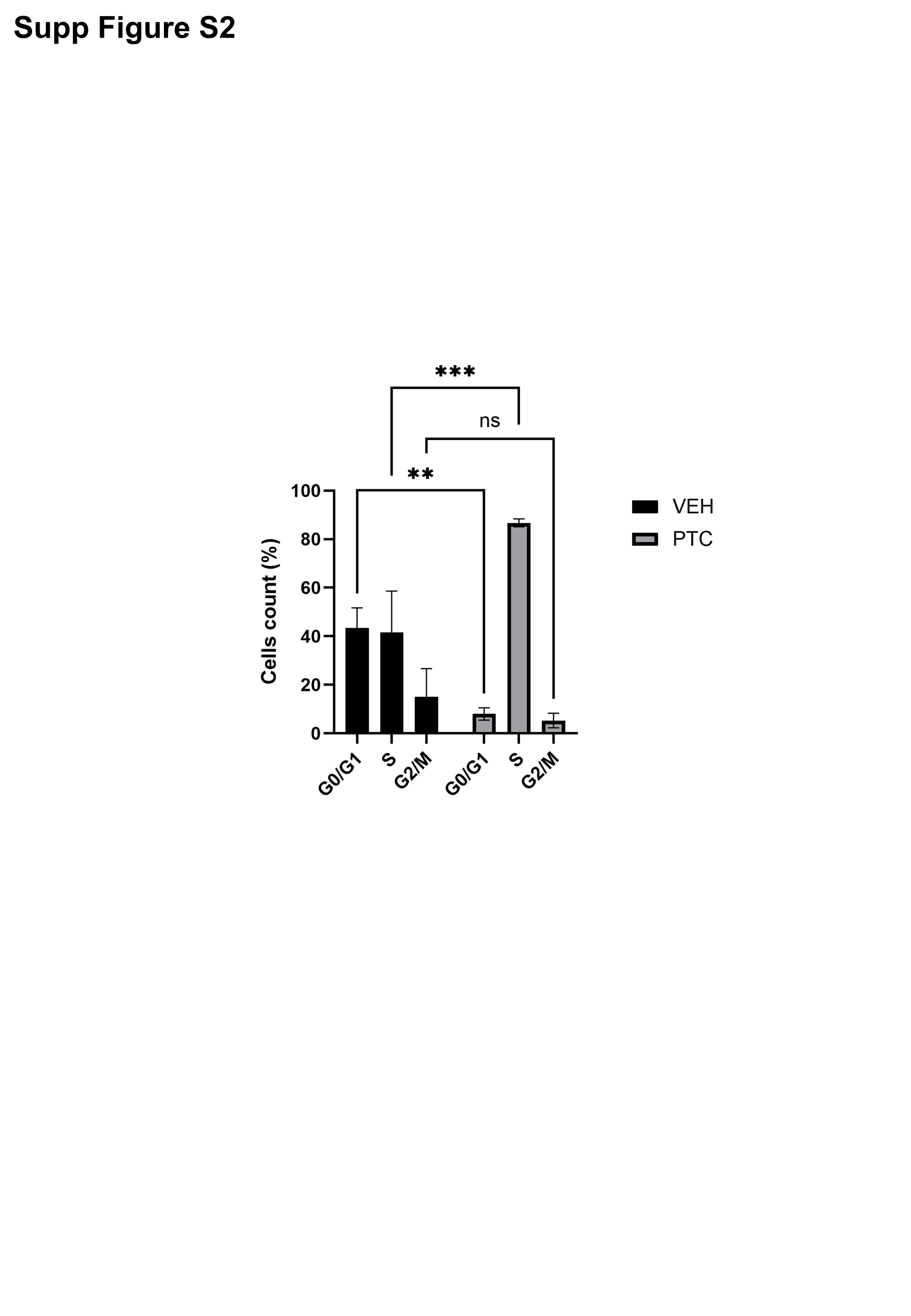

Supplement: Supplementary file 4 — Fig. S2 [file 41419_2026_8815_MOESM4_ESM.tif]

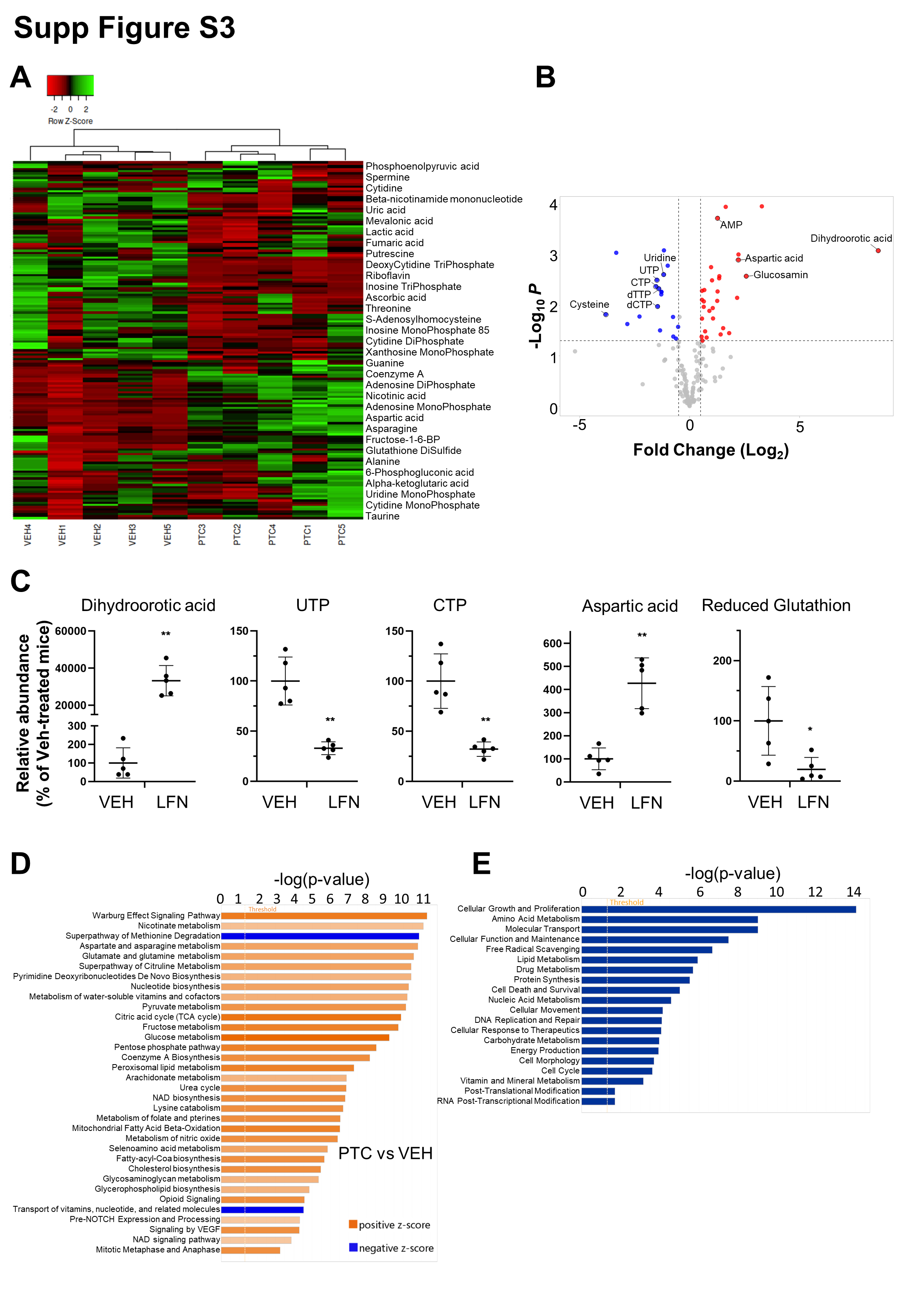

Supplement: Supplementary file 5 — Fig. S3 [file 41419_2026_8815_MOESM5_ESM.tif]

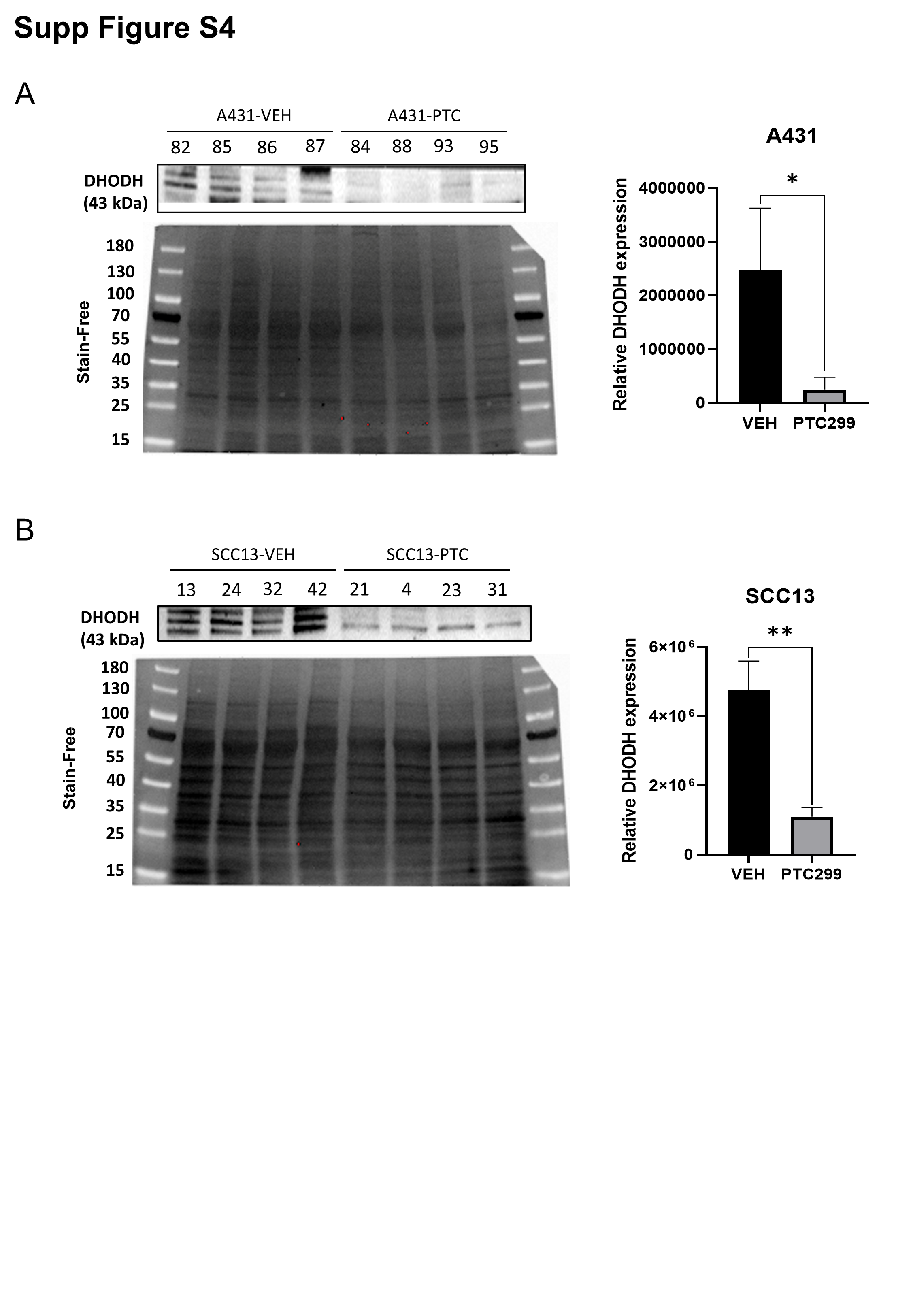

Supplement: Supplementary file 6 — Fig. S4 [file 41419_2026_8815_MOESM6_ESM.tif]
