# Supplementary material for: DHODH inhibition suppresses cutaneous squamous cell carcinoma growth by the induction of differentiation through perturbation of the cellular redox balance
Source: Cell Death Dis. 2026 Apr 28;17(1):566. doi: 10.1038/s41419-026-08815-w (PMC13260998; doi:10.1038/s41419-026-08815-w)
Supplement: Supplementary file 7 — full length western blots used in the manuscript [file 41419_2026_8815_MOESM7_ESM.pdf]

Supplementary Data 2:

Full length western blots used in the manuscript

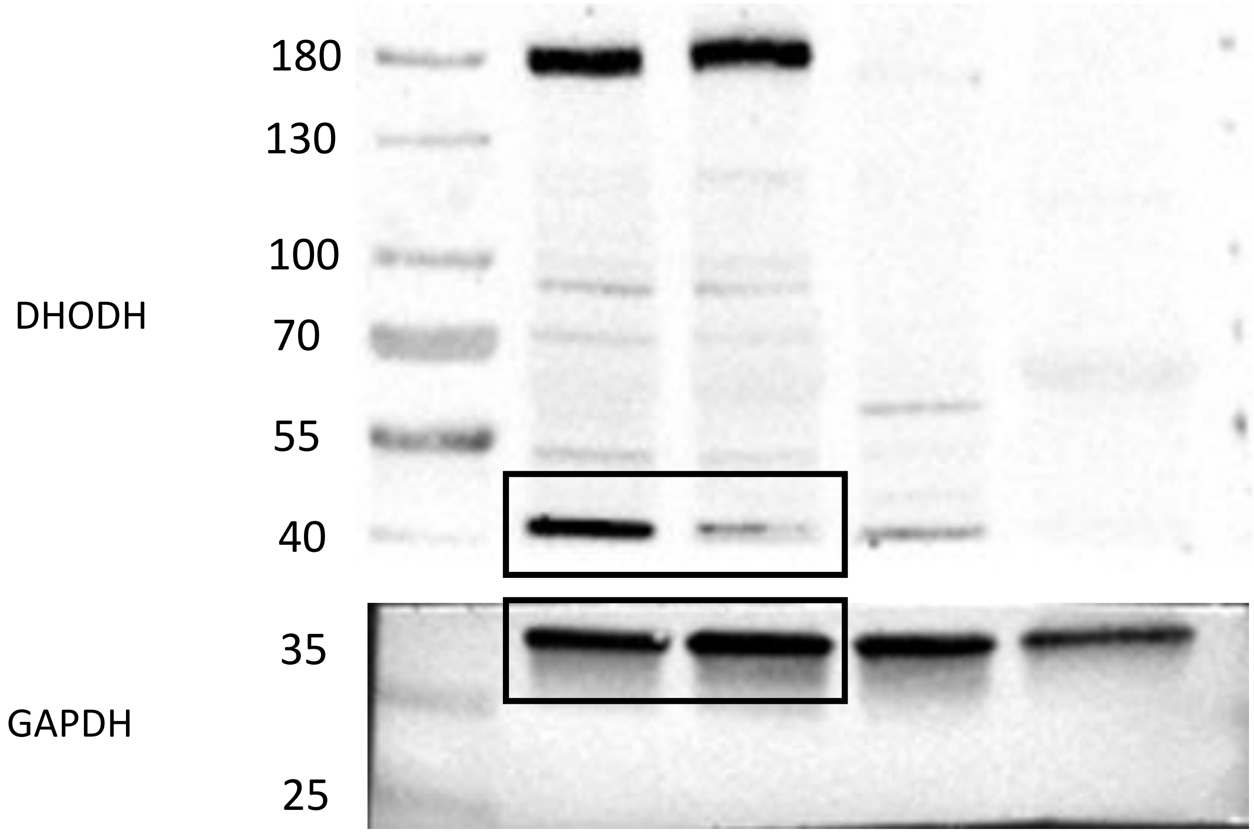

Figure S1. Full unedited gel used in Figure 1G.

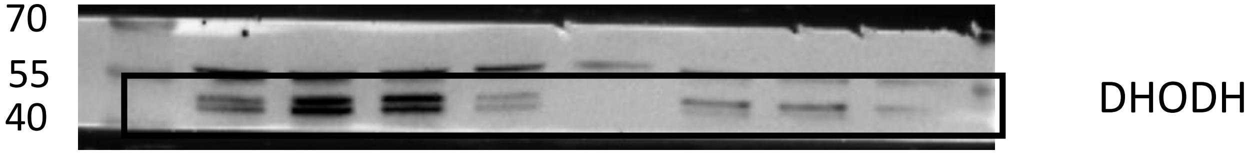

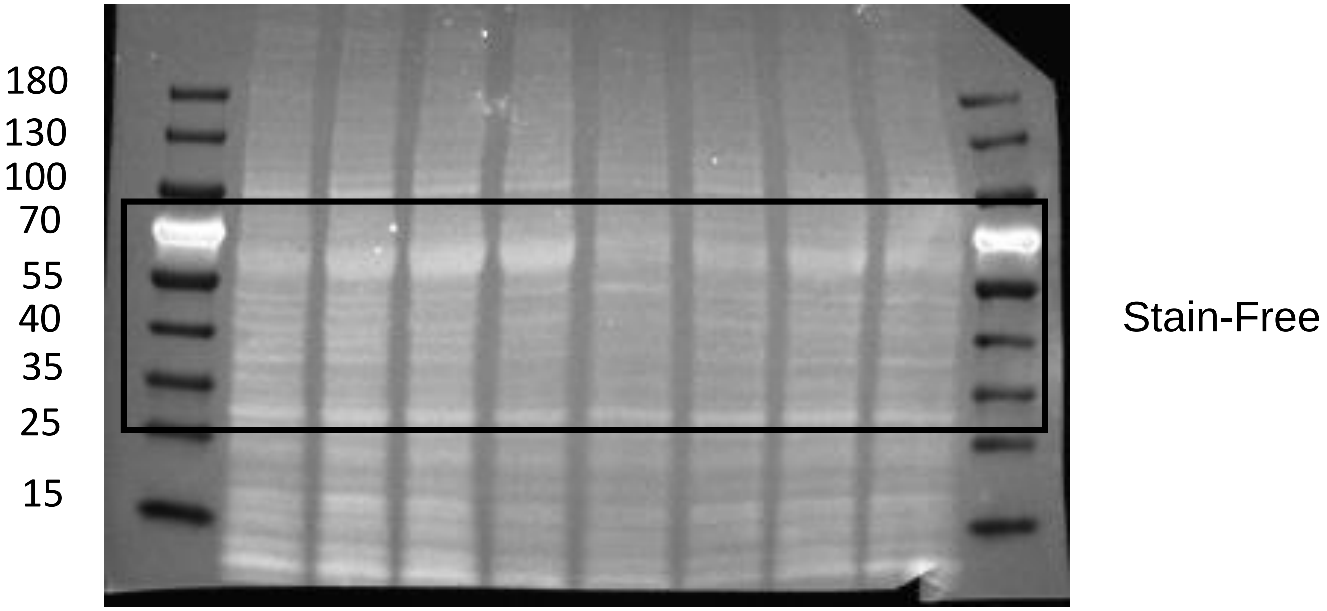

Figure S2. Full unedited gel used in Figure 1J.

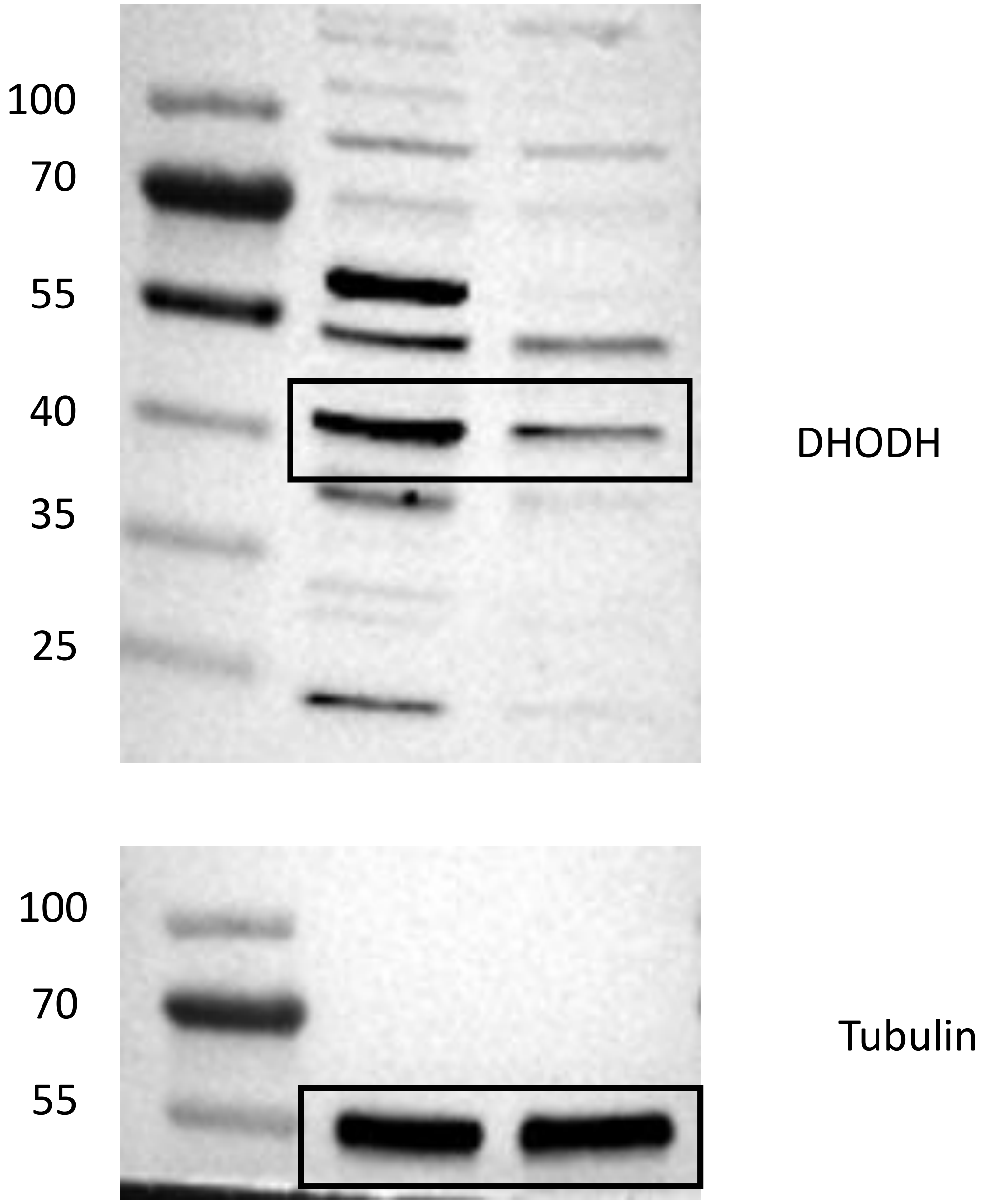

Figure S3. Full unedited gel used in Figure 2G.

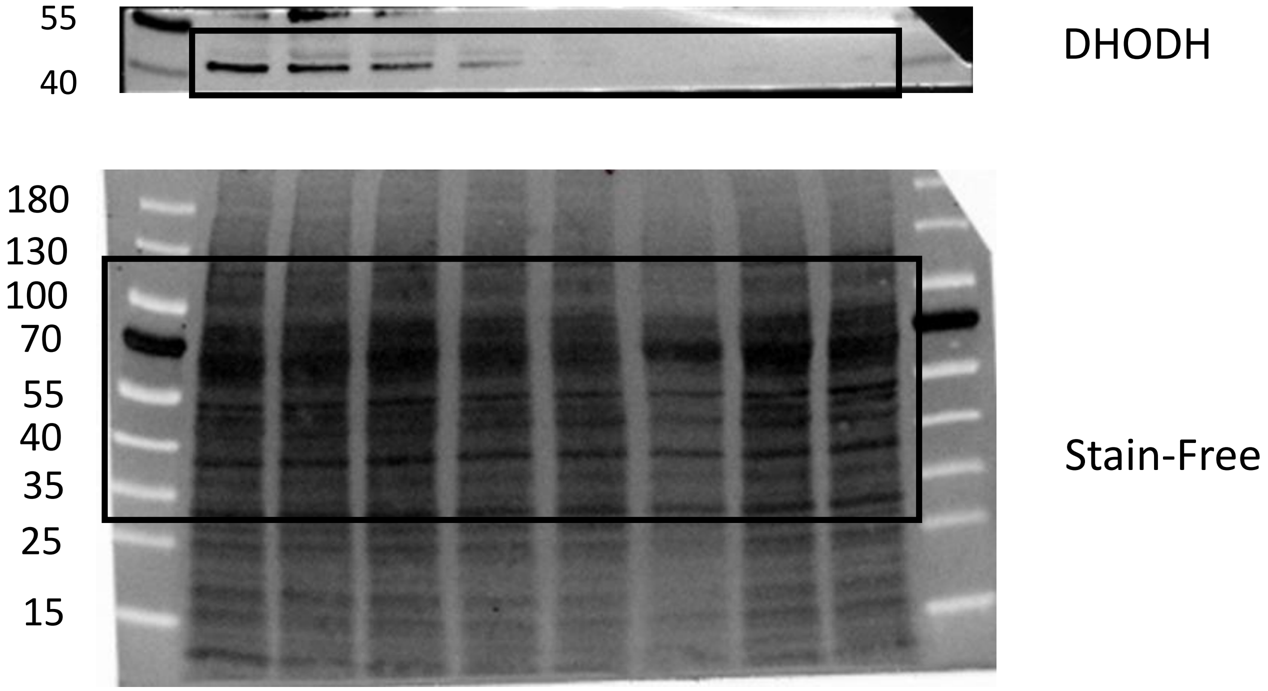

Figure S4. Full unedited gel used in Figure 2J.

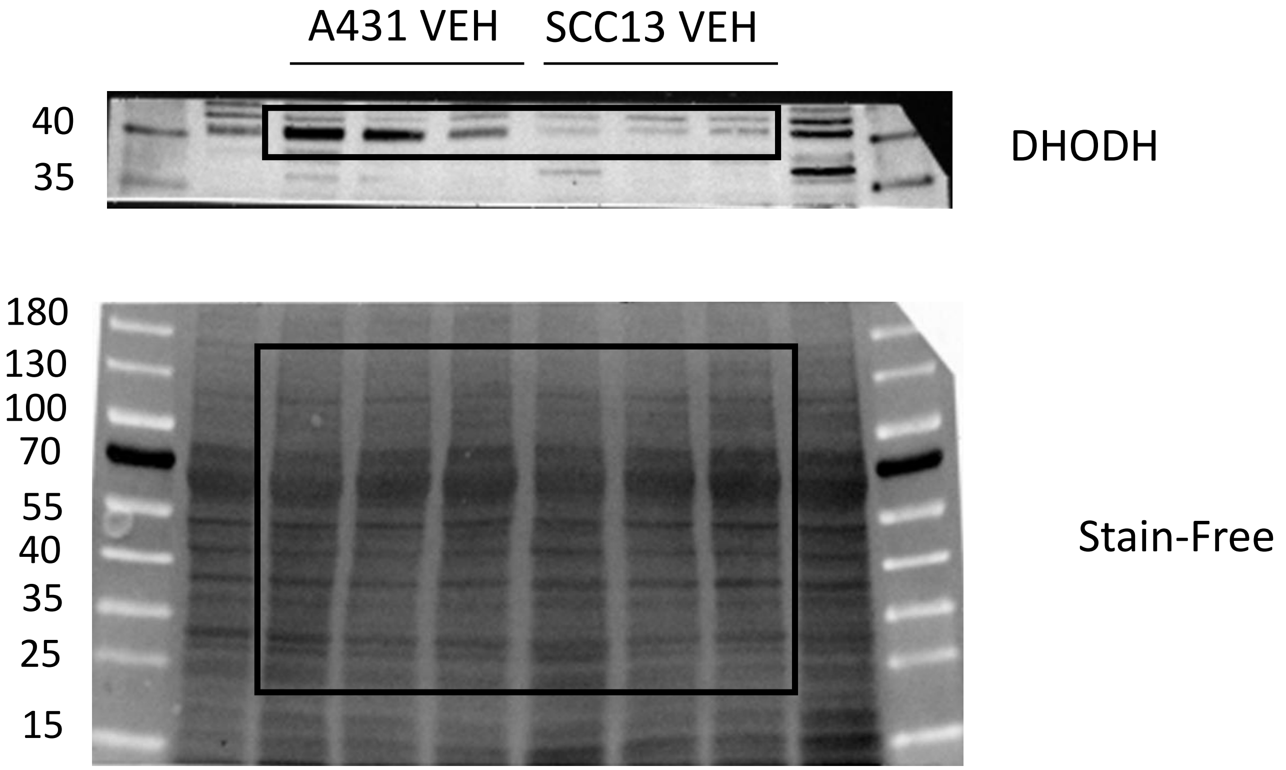

Figure S5. Full unedited gel used in Figure 3A.

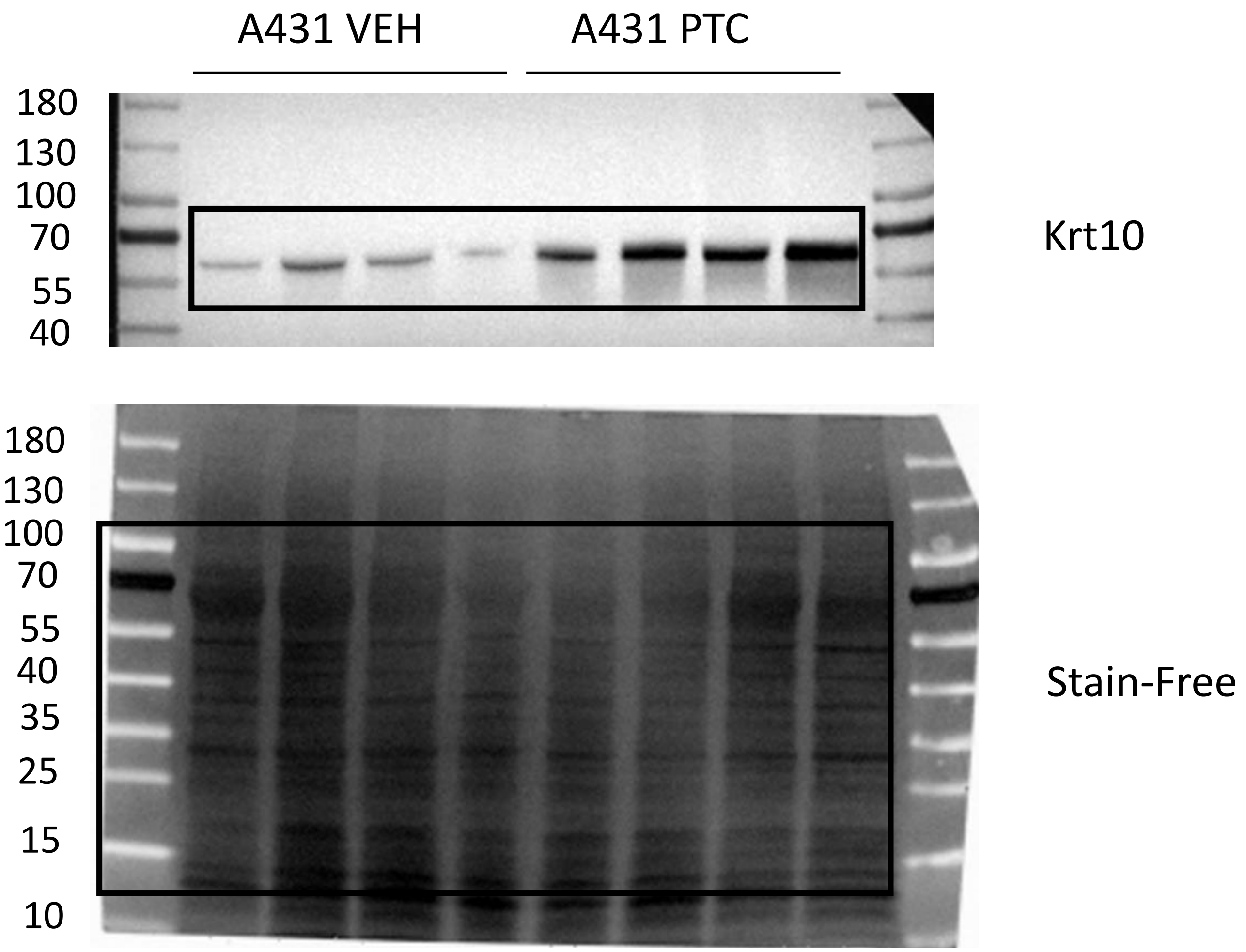

Figure S6. Full unedited gel used in Figure 8B.

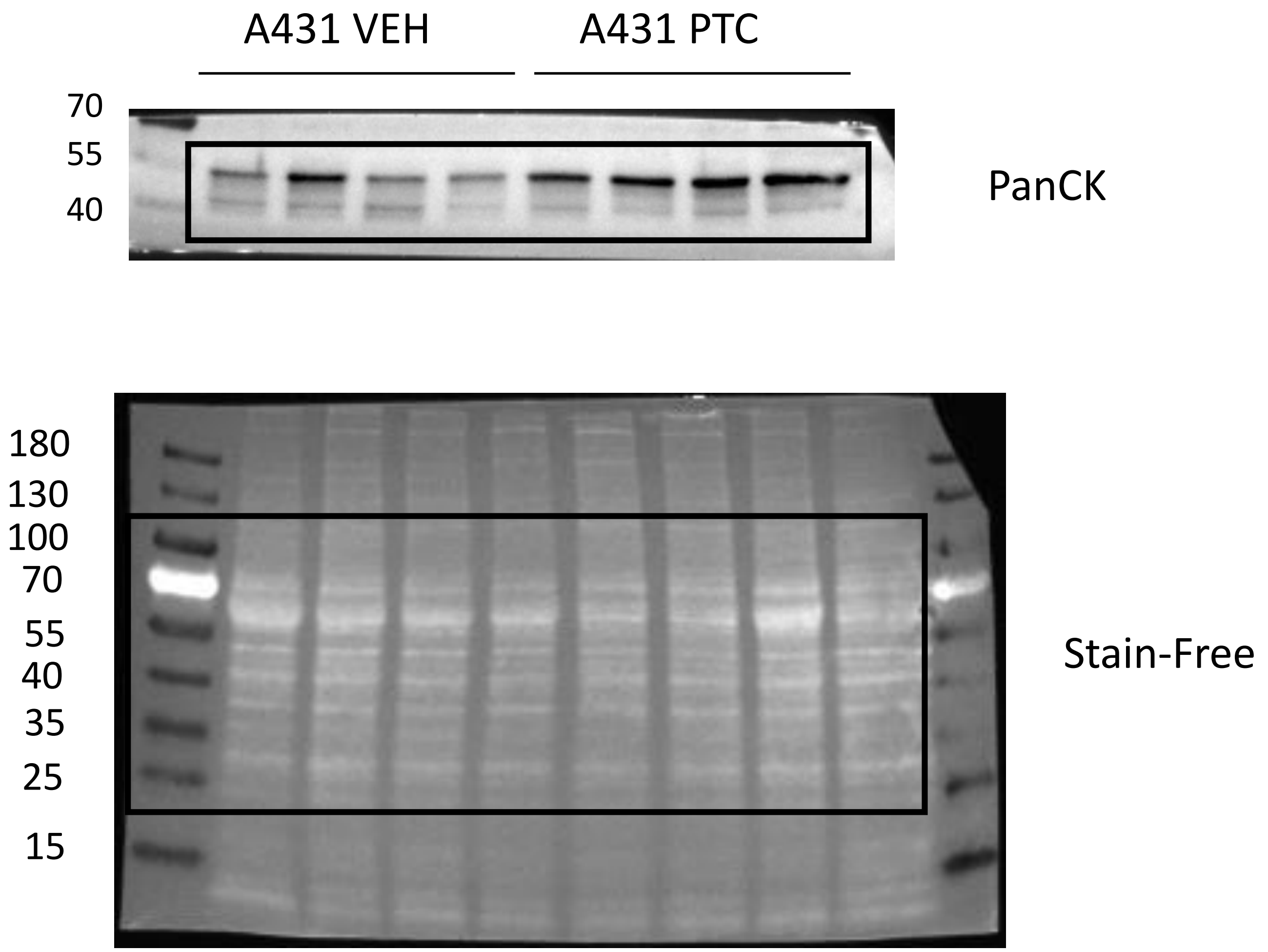

Figure S7. Full unedited gel used in Figure 8B.

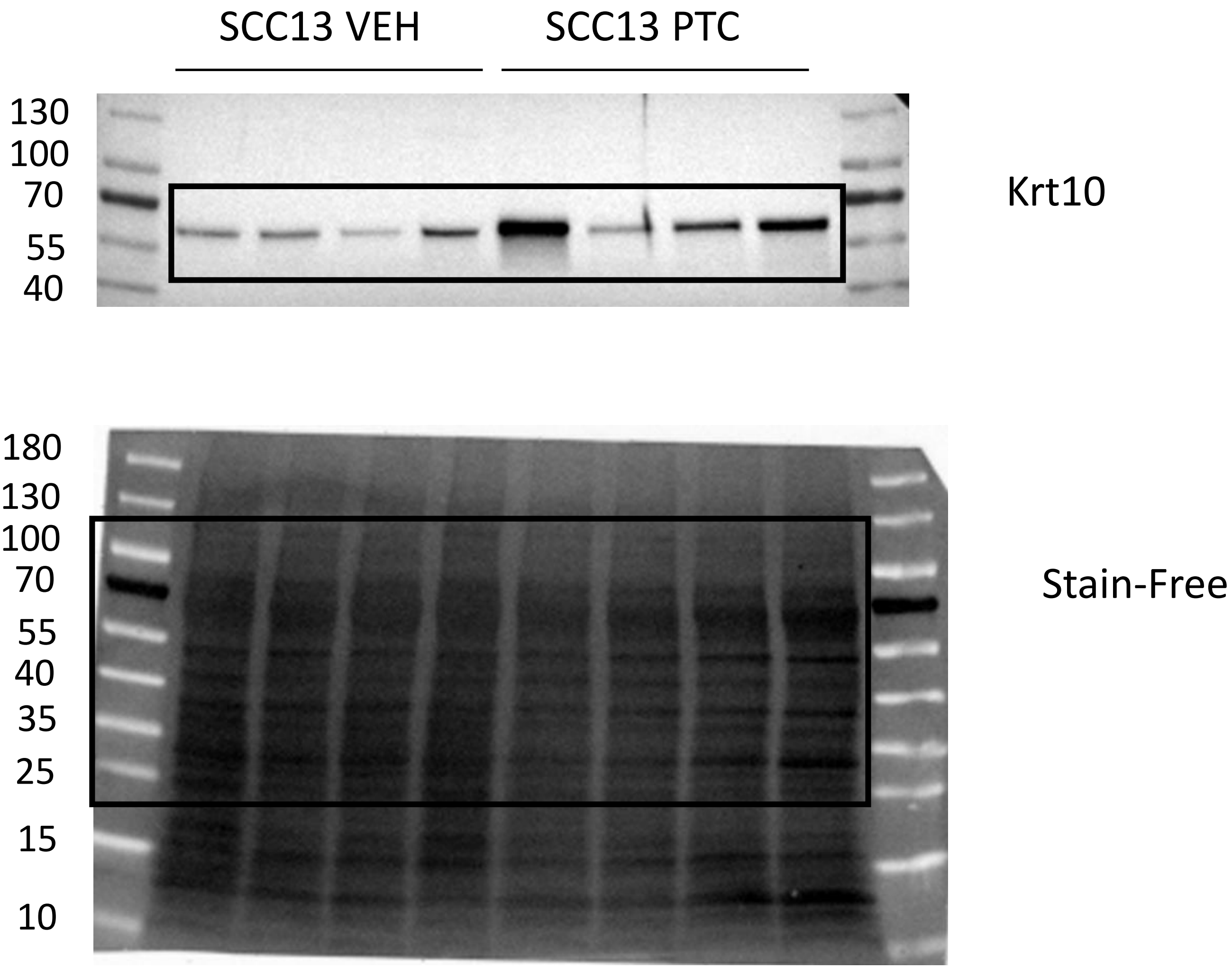

Figure S8. Full unedited gel used in Figure 8B.

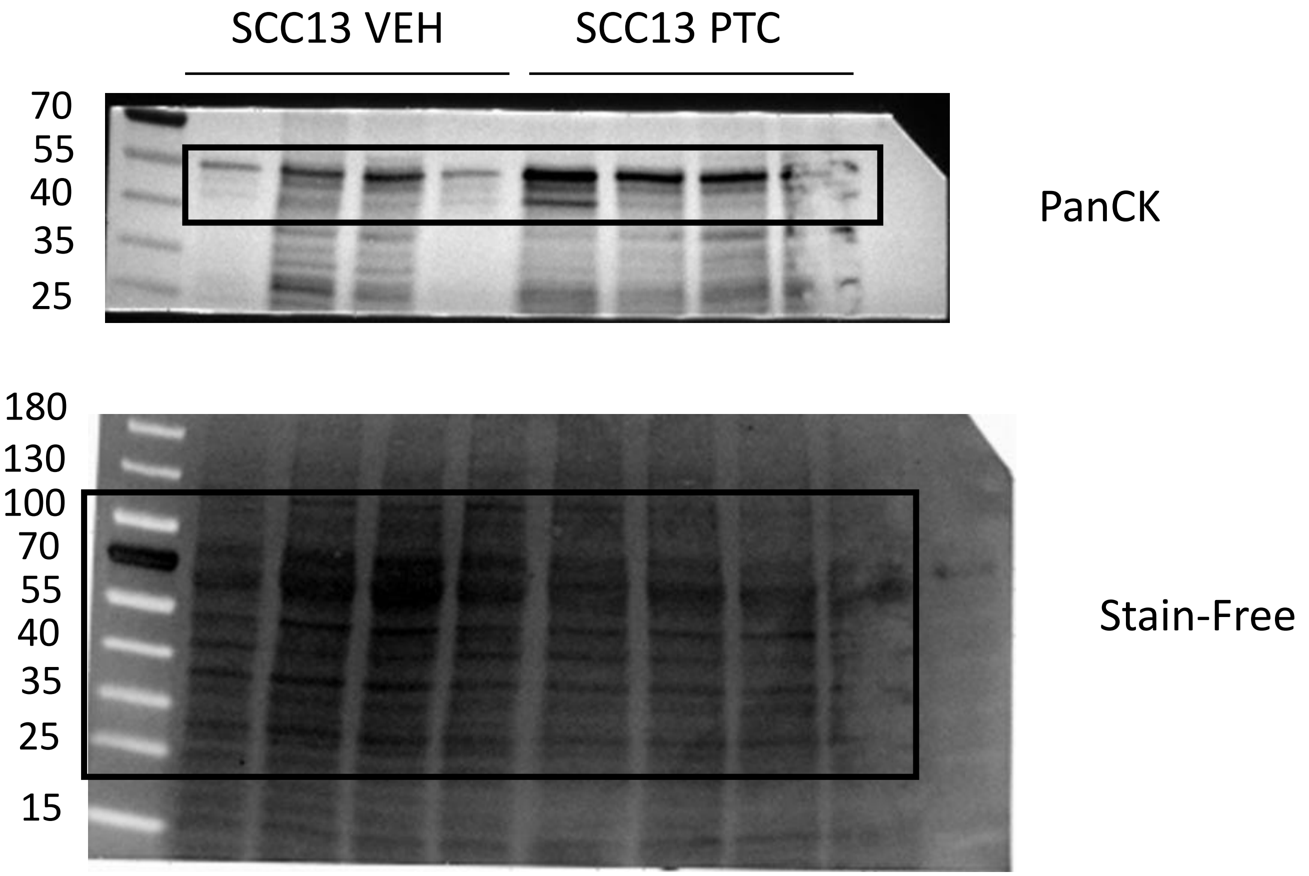

Figure S9. Full unedited gel used in Figure 8B.

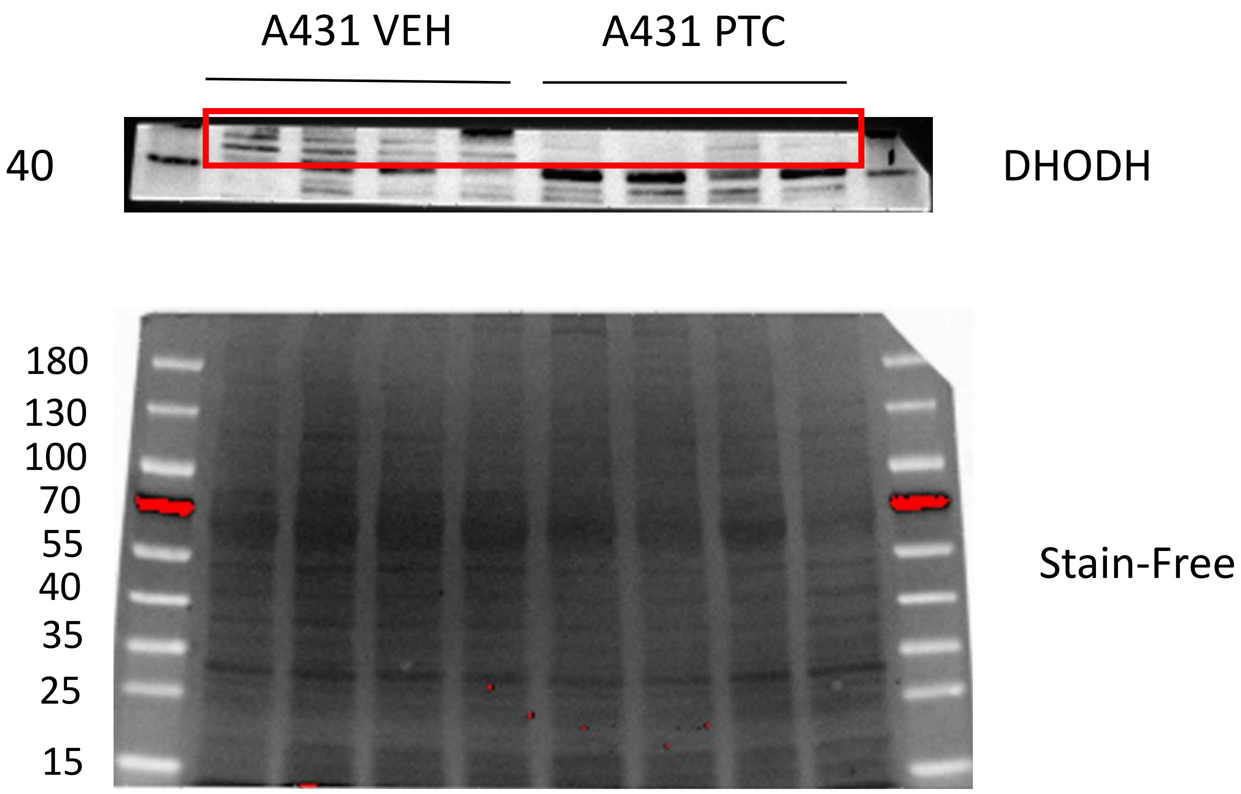

Figure S10. Full unedited gel used in SUPP Figure S4.

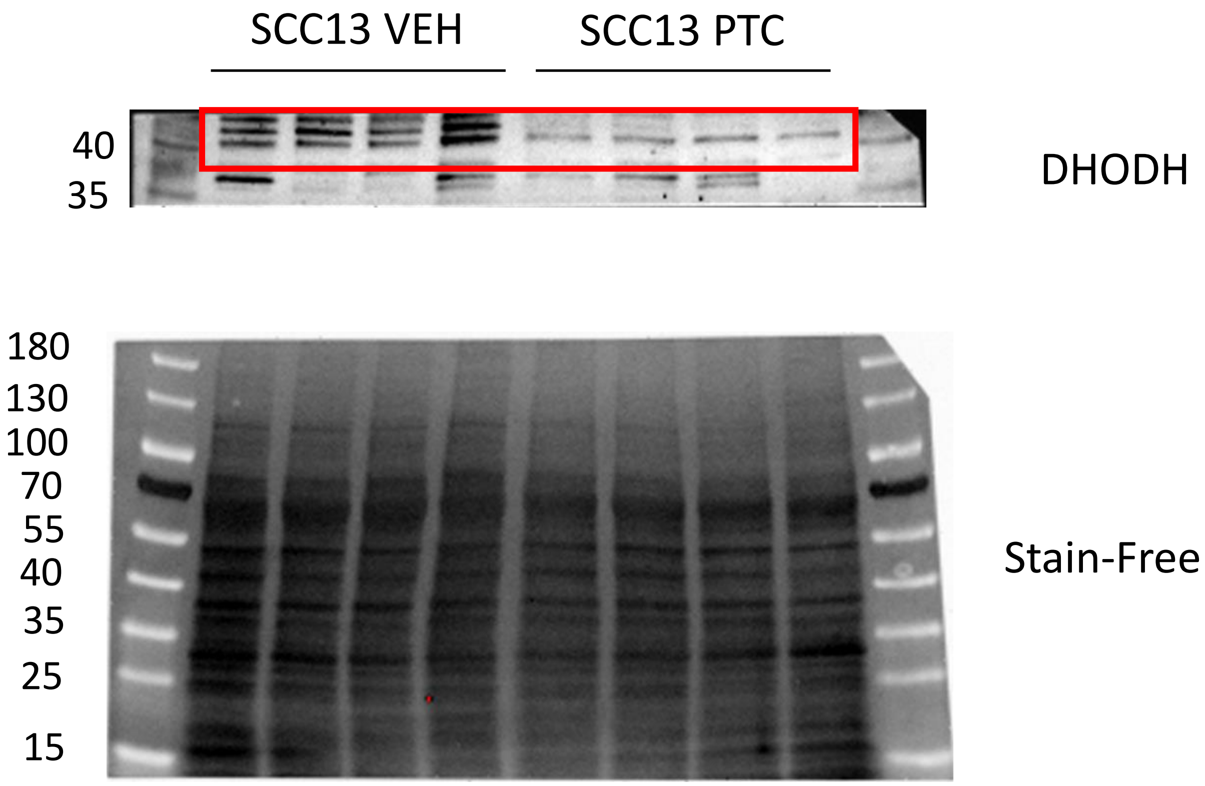

Figure S11. Full unedited gel used in SUPP Figure S4.
